# Effects of aerobic exercise interventions on cognitive function, sleep quality, and quality of life in older adults with mild cognitive impairment: a systematic review and meta-analysis

**DOI:** 10.3389/fneur.2025.1693052

**Published:** 2025-12-17

**Authors:** Wanyu Shu, Liang Chen, Jiadong Qiu, Sung Min Kim

**Affiliations:** Department of Sport Science, Hanyang University, Seoul, Republic of Korea

**Keywords:** aerobic exercise, mild cognitive impairment, cognitive function, sleep quality, quality of life

## Abstract

**Background:**

Aerobic exercise, as a non-pharmacological intervention, has been widely recognized for its potential benefits on cognitive function in individuals with mild cognitive impairment (MCI). However, systematic evidence regarding its effects on other critical health domains, such as sleep quality and quality of life, remains limited. Moreover, previous meta-analyses have typically included a relatively small number of randomized controlled trials (RCTs), which may constrain the generalizability and objectivity of their findings.

**Objective:**

This study aimed to evaluate the effects of aerobic exercise on cognitive function, sleep quality, and quality of life in older adults with MCI, and to identify key exercise prescription parameters based on the FITT principle (frequency, intensity, time, and type).

**Results:**

A total of 26 randomized controlled trials involving 2,085 individuals with MCI were included. The meta-analysis revealed that aerobic exercise had a statistically significant positive effect on global cognitive function (SMD = 0.81, 95% CI: 0.58–1.05, *p* < 0.00001) and quality of life (SMD = 1.26, 95% CI: 0.70–1.82, *p* < 0.00001). However, no significant improvement was observed in sleep quality (SMD = 0.07, 95% CI: −1.79–1.93, *p* = 0.94). Subgroup analysis further indicated that interventions conducted four times per week, lasting more than 50 min per session, at moderate intensity, and primarily involving walking were most effective in improving cognitive function.

**Conclusion:**

The findings of this study demonstrate that aerobic exercise may significantly improve cognitive function and quality of life in older adults with MCI, with enhanced effects observed when intervention parameters are optimized.

**Systematic review registration:**

https://www.crd.york.ac.uk/prospero/display_record.php?ID=CRD42024495979, Unique Identifier: CRD42024495979.

## 1 Introduction

Effective methods for delaying and controlling cognitive decline in older individuals are the focus of growing research as the global aging population is rapidly expanding (1). Currently, around 50 million people globally are estimated to have dementia according to the World Health Organization. Projections indicate that by 2050, this number is expected to triple (2). Dementia significantly affects the quality of life in later years, placing a considerable burden on families and society (3). Thus, dementia treatment and early detection are essential.

The MCI is commonly regarded as a transitional stage between normal cognitive function and dementia (4). The diagnostic criteria for MCI include subjective concerns about cognitive changes, impairment in one or more cognitive domains, preserved activities of daily living (ADL), and the absence of dementia (5, 6). In MCI research, global cognitive function refers to an individual’s overall cognitive performance across multiple domains, such as memory, attention, executive function, language, and visuospatial ability, and is typically assessed using standardized screening tools like the Mini-Mental State Examination (MMSE) or the Montreal Cognitive Assessment (MoCA) (4, 7). The prevalence of MCI in older adults is approximately 16.6%, and about 23.8 to 46% of elderly individuals with MCI progress to dementia within 3 years of diagnosis (8–10). Therefore, developing effective preventive strategies to delay or even reverse cognitive decline in MCI patients is of critical importance.

Interventions for the MCI phase encompass both pharmacological and non-pharmacological domains. Pharmacological interventions for MCI mainly include acetylcholinesterase inhibitors, the NMDA receptor antagonist memantine, and antihypertensive agents. However, current evidence remains insufficient to support the long-term efficacy of these drugs in improving cognitive function among individuals with MCI (11). Moreover, multiple systematic reviews and a scientific statement issued by the American Heart Association have consistently reported that acetylcholinesterase inhibitors, antihypertensive medications, statins, and nonsteroidal anti-inflammatory drugs (NSAIDs) do not reduce the incidence of dementia (12–15).

Given these limitations, non-pharmacological interventions have gained increasing attention as relatively safe and scalable strategies to preserve and enhance cognitive function in people with MCI (12). Non-pharmacological interventions for MCI include physical activity (PA), music therapy (MT), and computerized cognitive training (CCT), among other modalities (16–22). Physical activity is widely recognized for its therapeutic benefits as a non-pharmacological intervention.

Exercise is a subset of physical activity that is planned, structured, and purposeful, aimed at improving or maintaining health and fitness. In contrast, physical activity refers to any bodily movement produced by skeletal muscles that results in energy expenditure, such as household chores or recreational activities (23). Exercise plays a critical role in delaying the progression of MCI to dementia, particularly by significantly benefiting executive function and memory (24). Moreover, exercise incorporates cognitive and social components, which may further enhance overall brain function (25).

Aerobic exercise, characterized by sustained and rhythmic activities involving large muscle groups, aims to enhance aerobic fitness—the ability of the cardiovascular system to deliver oxygen to working muscles (26). Findings from a meta-analytic subgroup analysis suggest that aerobic exercise, compared with other forms of physical activity, may yield the most pronounced improvements in cognitive function among individuals with MCI (27). This type of exercise enhances cardiopulmonary function, increases cerebral blood flow, boosts cognitive reserve, and enlarges hippocampal volume, collectively supporting cognitive improvement (28–30). Additionally, aerobic exercise stimulates the production of brain-derived neurotrophic factor (BDNF), which enhances synaptic plasticity and neuronal density. These changes improve neuroplasticity and support cognitive performance (31–33). Furthermore, aerobic exercise is accessible, cost-effective, and low-risk, making it ideal for older adults (34).

Notably, recent research has increasingly highlighted the relationship between sleep disturbances and cognitive decline in individuals with MCI, underscoring the critical role of sleep quality in maintaining cognitive health. Several studies have reported that the prevalence of sleep disturbances among individuals with MCI ranges from 60.3 to 70.1%, which is significantly higher than that observed in cognitively healthy older adults (35–37). Moreover, over 60% of MCI patients have subjectively reported experiencing poor sleep quality (36). When poor sleep quality persists for more than 2 years, the risk of hippocampal atrophy and abnormal beta-amyloid accumulation markedly increases, accelerating the progression from MCI to dementia by approximately threefold (38, 39). Therefore, proactive management of sleep disturbances in older adults with MCI is of both theoretical importance and practical relevance.

Meanwhile, the impact of MCI on quality of life (QoL) remains a subject of debate (40). Some studies suggest that MCI does not significantly impair QoL, whereas others report that cognitive decline in individuals with MCI is often accompanied by varying degrees of deterioration in emotional regulation, social engagement, self-efficacy, and satisfaction with daily living (41, 42). Given that QoL is closely linked to a range of cognitive functions—such as attention, memory, and language—and that individuals with MCI typically exhibit impairments in these domains to varying extents (43, 81), it is essential to examine the relationship between cognitive decline and QoL in greater depth. Such analysis is crucial for developing effective assessment frameworks and intervention strategies tailored to the specific needs of this population.

Recently, Ahn and Kim (44) conducted a systematic review and meta-analysis examining the effects of aerobic exercise on cognitive function and sleep in older adults with MCI. While informative, that review included only 11 RCTs, covering cognition and just two studies on sleep, and did not assess quality of life (44). Similarly, Han et al. (4) focused primarily on cognition and sleep, but their analysis was based on a limited evidence pool of 18 RCTs (4).

Expanding upon these earlier reviews, the present study incorporates 26 RCTs published up to May 2025, including 8 additional trials not covered previously, of which 6 were newly published between 2024 and 2025. Notably, this study includes 24 RCTs on cognitive function, along with 5 on sleep quality and 7 on quality of life, thereby broadening the outcome scope. Importantly, the inclusion of the most recent trials resulted in a slightly lower but more stable effect size for global cognition, indicating an updated and more conservative estimate of the benefits of aerobic exercise in MCI. By integrating a larger and more diverse body of evidence, this meta-analysis offers greater statistical power and a more comprehensive evaluation of the multidimensional benefits of aerobic exercise in MCI.

In summary, several meta-analyses have examined the effects of aerobic exercise on cognitive function in older adults with MCI (44–46). However, these studies were generally limited by small sample sizes, restricted intervention types, and a predominant focus on cognitive outcomes. Although some also addressed sleep or quality of life measures, the supporting evidence for these domains remains relatively weak. Therefore, the present study systematically evaluates the effects of aerobic exercise on global cognition, sleep quality, and quality of life in older adults with MCI. It further explores how exercise characteristics, including modality, frequency, intensity, duration, and assessment tools, influence intervention outcomes. By synthesizing evidence from randomized controlled trials, this meta-analysis aims to provide updated and empirically grounded recommendations for optimizing exercise-based management strategies for MCI.

## 2 Materials and methods

### 2.1 Protocol and registration

This study was conducted after registration with the International Prospective Systematic Evaluation Registry database (PROSPERO), and registration ID: CRD42024495979, https://www.crd.york.ac.uk/prospero/display_record.php?ID=CRD42024495979. The study adhered to the Preferred Reporting Items for Systematic Reviews and Meta-Analysis (PRISMA) criteria.

### 2.2 Search strategy

As of May 2025, we conducted a comprehensive search across four major academic databases: Web of Science, Cochrane Library, PubMed, and EMBASE. The search strategy combined MeSH terms and free-text keywords related to mild cognitive impairment, aerobic exercise, and randomized controlled trials. For example, the core PubMed search string was: (“Cognitive Dysfunction”[Mesh] OR “Mild Cognitive Impairment”[Title/Abstract]) AND (“Exercise”[Mesh] OR “Aerobic Exercise”[Title/Abstract] OR “Physical Activity”[Title/Abstract]) AND (“Randomized Controlled Trial”[Publication Type] OR randomized[Title/Abstract]). The full search syntax for all databases, including detailed synonyms and Boolean operators, is provided in Appendix A. Two authors (WYS and LC) independently screened titles, abstracts, and full texts to identify relevant studies. Disagreements were resolved through discussion or consultation with a third arbitrator (JDQ).

### 2.3 Inclusion and exclusion criteria

Inclusion criteria: (1) participants: aged 60 years or older and diagnosed with MCI, including both amnestic MCI (aMCI) and non-amnestic MCI (naMCI), based on validated diagnostic criteria such as Petersen’s criteria. (2) Interventions: Evidence from previous studies indicates that at least 6 weeks of aerobic exercise is required to yield significant benefits for older adults with MCI (47). Accordingly, the intervention group participated in aerobic exercise lasting at least 6 weeks, with a frequency of at least once per week. (3) Comparisons: No specific exercise intervention was administered to the control group, only maintaining their daily activities and health education, including stretching, balance training, or movement education. (4) Outcomes: Global cognitive function, Sleep Quality and Quality of life. (5) Design: For patients with MCI, this study comprises all published RCTs on aerobic exercise as an exercise intervention.

Excluded criteria: (1) Patients without MCI. (2) Non-aerobic exercise intervention was given to the intervention group. (3) The control group underwent an exercise intervention. (4) Non-RCTs or animal studies. (5) Unavailable studies, such as reviews, meta-analyses, abstracts, study protocols and case reports. (6) The original data could not be located. (7) Gray literature, including dissertations, conference proceedings, or unpublished reports, and non-English language studies were excluded to ensure data quality, methodological consistency, and replicability across included trials.

### 2.4 Data extraction

Two authors, SWY and CL, independently extracted data for each included study. We extracted the following information from each study: study information (authors, country, year and sample size), participant characteristics (gender and mean age), intervention group characteristics (FITT-VP: Frequency, Intensity, Time, Type, Volume, and Progression), characteristics of the control group and outcome measures.

### 2.5 Risk of bias assessment

Authors (WYS and LC) independently used the revised Cochrane Risk of Bias Tool 2.0 (48) to assess bias risk in selected studies across five domains: randomization process, intervention deviations, missing data, outcome measures, and reporting options. They rated each domain as low risk, some concerns, or high risk. Disagreements were resolved through discussion or consultation with a third arbitrator (JDQ). Although inter-rater agreement (e.g., Cohen’s *κ*) was not statistically calculated, consensus was reached on all final judgments after independent evaluation to ensure methodological rigor and reliability.

### 2.6 Statistical analyses

Data were analyzed by Review Manager 5.4 and Stata 17.0 statistical software, following the guidelines outlined in the Cochrane Handbook (49). Meta-analyses calculated changes from baseline to post-intervention using mean and standard deviation (SD). The effect size was measured using mean difference (MD) or standardized difference (SMD), Standardized Mean Difference (SMD) was used to estimate pooled effect sizes when assessing outcome measures using different scales of measurement. Effect sizes were defined as small (0.2), medium (0.5), or large (0.8) (50). Data summaries included 95% confidence intervals (CI). Study heterogeneity was evaluated with the *I*^2^ index, considered low at *I*^2^ ≤ 25%. Moderate heterogeneity was determined when *I*^2^ was ≤50% and >25%. *I*^2^ > 50% indicated high heterogeneity (51).

This study conducted subgroup analyses of variables to identify effective exercise strategies for patients with MCI based on the FITT principle and to examine potential sources of heterogeneity in cognitive function. Analyzed variables included 11 types of interventions (e.g., aerobic dance, walking, Tai Chi), 3 levels of intensity (low, moderate, and moderate-to-high), 2 durations (≥50 min and <50 min), 5 frequencies (once to five times per week), and 10 assessment tools (e.g., MoCA, MMSE, and ADAS). The categorization of all variables was based on the information reported in the included studies.

## 3 Results

### 3.1 Study selection

**Figure 1:**
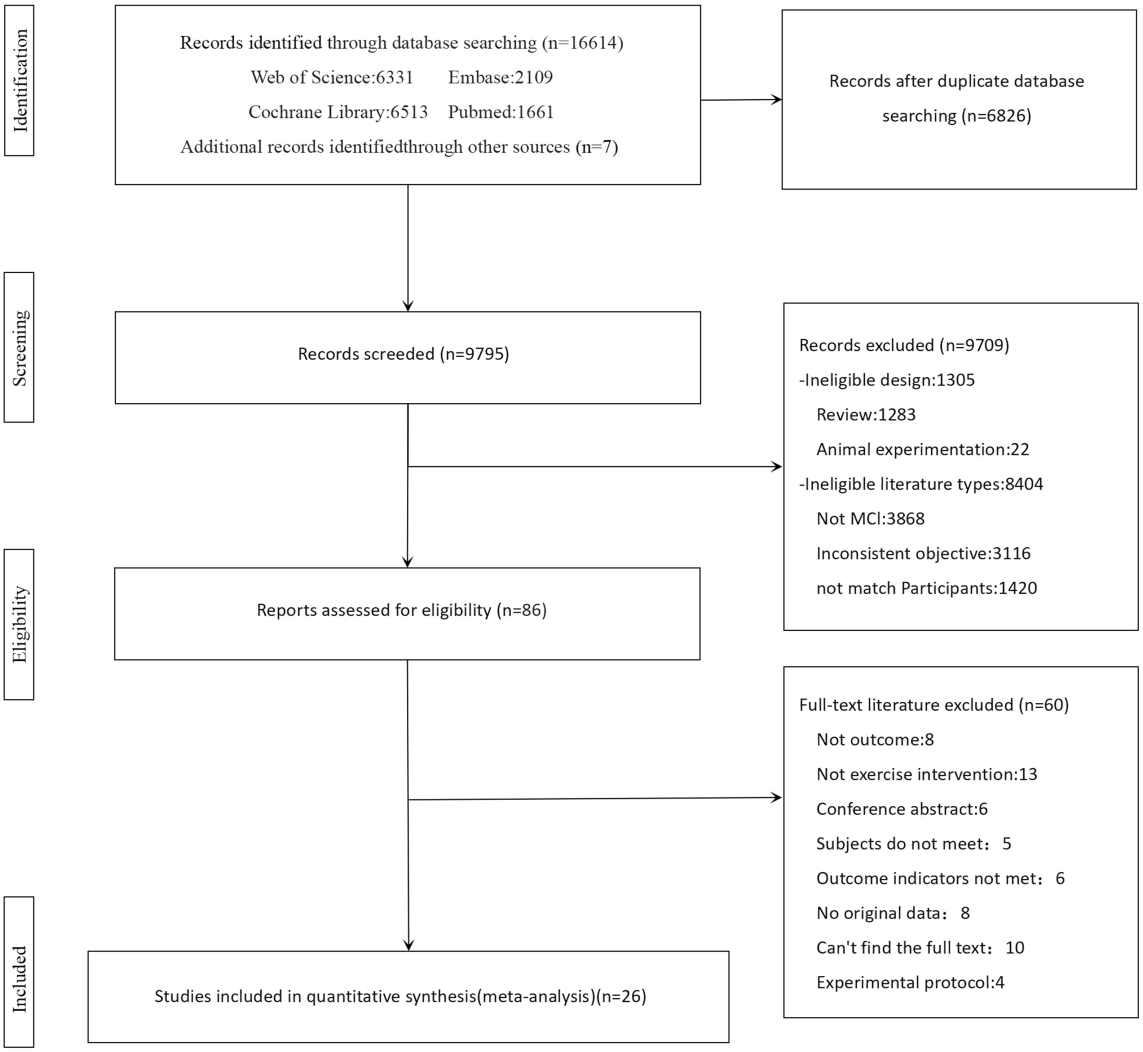
Study selection process flowchart.

Following the predefined search strategy, 16,614 articles were identified from four major electronic databases. Additionally, 7 articles were identified through reverse citation search, bringing the total to 16,621 articles. After removing 6,826 duplicates, 9,795 articles remained. After reviewing abstracts and titles, two reviewers (WYS and LC) narrowed down the field to 86 eligible studies, having excluded 9,709. Upon a thorough examination of the full-text papers, the study was conducted on the final 26 articles eligible for inclusion following exclusion of 60 studies. The screening process for eligible studies is illustrated in Figure 1.

### 3.2 Characteristics of included studies

Table 1 outlines the characteristics of the 26 randomized controlled trials included in this meta-analysis. The study involved a total of 2,085 participants diagnosed with MCI, all aged 60 years or older, including 1,031 in the intervention groups and 1,054 in the control groups. Specifically, for each outcome domain, global cognitive function analyses included 957 intervention and 982 control participants, sleep quality analyses included 174 intervention and 174 control participants, and quality of life analyses included 301 intervention and 288 control participants.

**Table 1:** Study characteristics.

No.	First author (year)	Country (region)	Sample size (I: C)	Age (mean ± SD)	Gender (female: male)	FITT	VP	Duration (weeks)	Comparison	Outcomes
1	Yu et al. (2022) (66)	Hong Kong	10:12	I (67.3 ± 4.2) C (67.6 ± 8.1)	I (7:3) C (10:2)	F: 3/week; I: Moderate T: 60 min; T: Tai Chi	V: 180 min P: Stable	24	Social activities	MoCA, PSQI SF12
2	Choi and Lee (2019) (68)	Korea	30:30	I (77.27 ± 4.37) C (75.37 ± 3.97)	I (25:5) C (26:4)	F: 2/week; I: NR; T: 60 min T: Virtual Kayak Paddling	V: 120 min P: Stable	6	Home exercise	MoCA
3	Song and Doris (2019) (29)	China	60:60	I (76.22 ± 5.76) C (75.33 ± 6.78)	I (48:12) C (42:18)	F: 3/week; I: Moderate T: 60 min; T: Stepping	V: 180 min P: Gradual	16	Health education	MoCA QOL-AD-C
4	Song et al. (2024) (56)	China	45:44	I (76.71 ± 5.96) C (75.20 ± 6.63)	I (36:9) C (32:12)	F: 3/week; I: Moderate T: 60 min; T: Aerobic Dancing	V: 180 min P: Stable	16	Health education	MOCA
5	Li et al. (2022) (69)	America	22:24	I (74.5 ± 5.6) C (74.9 ± 6.3)	I (8:14) C (15:9)	F: 2/week; I: NR T: 60 min; T: Tai Chi	V: 120 min P: Gradual	16	Stretching exercises	MoCA
6	Khatta et al. (2022) (73)	Pakistan	29:30	62.49 ± 1.82	24:35	F: 5/week; I: Moderate T: 60 min; T: Walking	V: 300 min P: Stable	6	Social activities	MMSE MoCA
7	Wang et al. (2024) (80)	China	30:30	60–69	NR	F: 4/week; I: NR; T: 40 min T: Chinese Square Dance	V: 160 min P: Stable	12	Social activities	MoCA
8	Lazarou et al. (2017) (74)	Greece	66:63	I (65.89 ± 10.76) C (67.92 9.47)	I (53:13) C (48:15)	F: 2/week; I: NR T: 60 min; T: Dancing	V: 120 min P: Gradual	40	Social activities	MMSE
9	Chang et al. (2021) (52)	China	62:47	I (76.56 ± 3.60) C (75.94 ± 3.61)	NR	F: 3/week; I: Moderate-to-high T: 30 min; T: Chinese Square Dance	V: 90 min P: Stable	18	Social activities	MoCA SF-12
10	Bademli et al. (2019) (75)	Turkey	30:30	I (72.24 ± 7.16) C (70.67 ± 8.34)	I (18:12) C (17:13)	F: 4/3/7/week; I: Moderate T: 80 min; T: Walking	V: 364 min P: Alternating frequency cycle	20	Social activities	SMMSE
11	Lam et al. (2014) (64)	Hong Kong	96:169	I (77.2 ± 6.3) C (78.3 ± 6.6)	NR	F: ≥3/week; I: NR T: ≥30 min; T: Tai Chi	V: 90 min P: Incremental	48	Stretching and relaxation exercises	MMSE ADAS
12	Lin et al. (2025) (54)	China	18:18	I (86.17 ± 6.022) C (81.96 ± 6.12)	I (9:9) C (13:5)	F: 3/week; I: NR T: 30 min; T: Tai Chi	V: 90 min P: Stable	12	Routine care	MoCA
12	Lin et al. (2025) (54)	China	18:18	I (85.28 ± 4.650) C (81.96 ± 6.12)	I (14:4) C (13:5)	F: 3/week; I: NR T: 30 min; T: Walking	V: 90 min P: Stable	12	Routine care	MoCA
13	Kohanpour et al. (2017) (70)	Iranian	10:10	67.85 ± 3.89	I (0:10) C (0:10)	F: 3/week; I: Moderate-to-high T: 21–39 min; T: Runing	V: 90 min P: Gradual	12	Placebo	MMSE
14	Qi et al. (2019) (55)	China	16:16	I (70.6 ± 6.2) C (69.1 ± 8.1)	I (11:5) C (12:4)	F: 3/week; I: Moderate T: 35 min; T: SDMIAD	V: 105 min P: Stable	12	Routine care	MMSE MoCA
15	Sánchez-Alcalá et al. (2024) (62)	Spain	47:45	I (71.43 ± 2.97) C (72.24 ± 2.92)	I (29:18) C (29:16)	F: 2/week; I: Moderate T: 60 min; T: Aerobic Dance	V: 120 min P: Incremental	12	Social activities	PSQI SF-36(CSF) SF-36(CSM)
16	Sánchez-Alcalá et al. (2025) (61)	Spain	47:45	I (71.43 ± 2.97) C (72.24 ± 2.92)	I (29:18) C (29:16)	F: 2/week; I: Moderate T: 60 min; T: Aerobic Dance	V: 120 min P: Incremental	12	Social activities	MMSE
17	Siu and Lee (2018) (65)	Hong Kong	80:80	≥60	I (58:22) C (60:20)	F: 2/week; I: Moderate T: 60 min; T: Tai Chi	V: 120 min P: Stable	16	Routine care	CMMSE
18	Khanthong et al. (2021) (71)	Thailand	35:36	I (60.26 ± 5.67) C (61.47 ± 7.49)	I (31:4) C (25:11)	F: 3/week; I: NR T: 60 min; T: Ruesi Dadton	V: 180 min P: Stable	12	Social activities	MoCA
19	Gao et al. (2024) (53)	China	27:27	I (78.6 ± 7.0) C (80.9 ± 8.4)	I (18:9) C (19:8)	F: 3/week; I: NR T: 50 min; T: Tai Chi	V: 150 min P: Stable	12	Routine care	ActiGraph QOL-AD
20	Varela et al. (2012) (63)	Spain	17:15	I (79.24 ± 10.07) C (79.4 ± 6.72)	27:21	F: 3/week; I: Low T: 30 min; T: Bike	V: 90 min P: Gradual	12	Recreational activities	MMSE
20	Varela et al. (2012) (63)	Spain	16:15	I (76.44 ± 11.38) C (79.4 ± 6.72)	27:21	F: 3/week; I: Moderate T: 30 min; T: Bike	V: 90 min P: Gradual	12	Recreational activities	MMSE
21	Wang et al. (2020)(57)	China	33:33	I (81.06 ± 5.17) C (81.09 ± 7.44)	I (26:7) C (21:12)	F: 3/week; I: Moderate; T: 40 min T: Chinese Square Dancing	V: 120 min P: Stable	12	Routine care	MoCA, MMSE SF-12(PCS) SF-12(MCS)
22	Doi et al. (2017)(72)	Japanese	67:67	I (75.7 ± 4.1) C (76.0 ± 4.9)	I (34:33) C (31:36)	F: 1/week; I: NR T: 60 min; T: Dance	V: 60 min P: Stable	40	Health education	MMSE
23	Choi and Lee (2018) (67)	Korea	30:30	I (74.90 ± 5.10) C (74.23 ± 4.38)	I (24:6) C (25:5)	F: 2/week; I: NR T: 60 min; T: GKP	V: 120 min P: Stable	6	Home exercise	MoCA
24	Wei and Ji (2014) (58)	China	30:30	I (66.73 ± 5.48) C (65.27 ± 4.63)	I (9:21) C (11:19)	F: 5/week; I: Moderate T: 30 min; T: Handball	V: 150 min P: Stable	24	Original life entertainment	MMSE
25	Zhao et al. (2021) (59)	China	31:32	I (73.35 ± 5.10) C (71.25 ± 6.73)	I (26:5) C (26:6)	F: 3/week; I: Moderate T: 60 min; T: Square Dancing	V: 180 min P: Stable	12	Health education	MoCA-P
26	Zhu et al. (2018) (60)	China	29:31	I (70.3 ± 6.7) C (69.0 ± 7.3)	I (15:14) C (21:10)	F: 3/week; I: Moderate T: 35 min; T: Aerobic Dance	V: 105 min P: Stable	24	Routine care	MoCA, SF-36

ADAS, Alzheimer’s Disease Assessment Scale; CMMSE, Chinese version of Mini-Mental State Examination; C, control group; FITT, Frequency, Intensity, Time, Type; GKP, Ground Kayak Paddling; I, Intervention group; MMSE, Mini Mental State Examination; MoCA, Montreal Cognitive Assessment; MoCA-P, Montreal Cognitive Assessment-Peking version; NR, No Response; QOL-AD-C, Quality of Life–Alzheimer’s disease, Chinese version; SDMIAD, Specially Designed Moderate-Intensity Aerobic Dance; SF-12, Short-Form 12 Health Survey; SF-36, 36-item Short Form Health Survey; SF-12(MCS), Short-Form 12 health survey mental component summary; SF-12(PCS), Short-Form 12 health survey physical component summary; SF-36(CSF), 36-item Short Form Health Survey Physical Component Summary; SF-36(CSM), 36-item Short Form Health Survey Mental Component Summary; SMMSE, Standardized Mini Mental State Examination; VP, volume, progression.

These studies were conducted in China (11 studies) (29, 52–60, 80), Spain (3) (61–63), Hong Kong (3) (64–66), Korea (2) (67, 68), One study each was conducted in the United States (69), Iran (70), Thailand (71), Japan (72), Pakistan (73), Greece (74), and Turkey (75). The sample sizes ranged from 20 to 265 participants. All included studies explicitly stated the eligibility criteria for participant recruitment.

The studies explored various aerobic exercises, including dance (11 studies), tai chi (6), walking (3), kayaking (2), and one study each in running, stepping, bike, handball, and traditional Thai exercise. Aerobic intervention durations ranged from 21 to 80 min, with exercise frequencies between once and five times weekly, and cycles lasting 6 to 48 weeks. In contrast, the control groups in these trials were compared to a variety of activities, such as social activities, stretching exercises, health education, placebo, and routine care.

The outcome measures included global cognitive function, sleep quality and quality of life. A total of 24 studies assessed global cognitive performance using standardized tools such as the Mini-Mental State Examination (MMSE), the Montreal Cognitive Assessment (MoCA), and the Alzheimer’s Disease Assessment Scale–Cognitive Subscale (ADAS-Cog). Sleep quality was evaluated in 5 studies using the Pittsburgh Sleep Quality Index (PSQI) and ActiGraph monitoring. Quality of life was assessed in 7 studies employing instruments such as the Quality of Life in Alzheimer’s Disease Scale (QOL-AD) and the 12-Item Short Form Health Survey (SF-12).

### 3.3 Bias risk of the included studies

**Figure 2:**
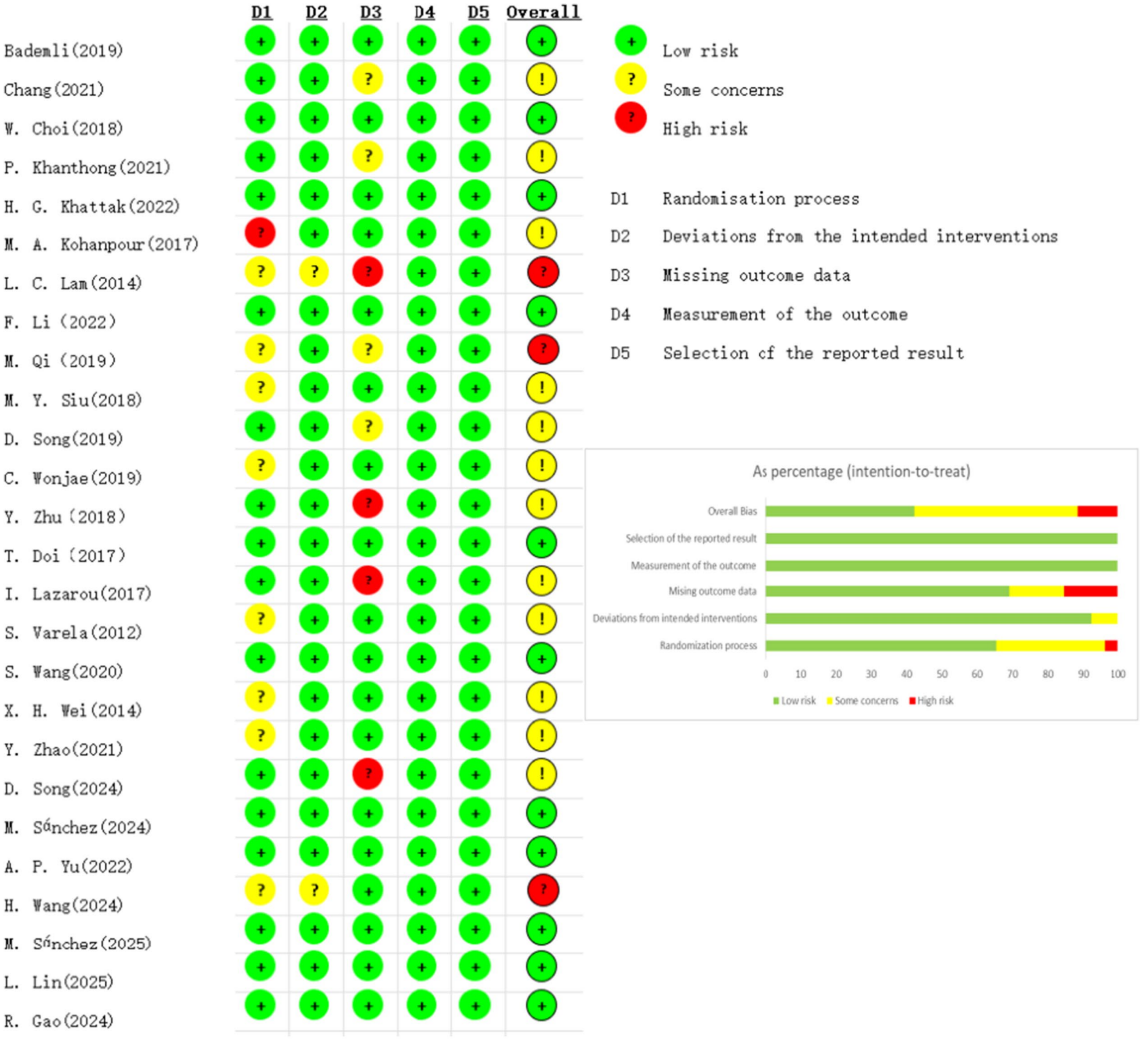
Risk of bias assessment.

The Cochrane Risk of Bias Tool was used to assess the risk of bias in the 26 included randomized controlled trials (Figure 2). Of these, 11 trials were rated as having a “low risk” of bias, 12 trials were rated as having “some concerns,” and 3 trial was rated as “high risk.” Domain-specific analysis indicated that the majority of studies rated as having “some concerns” showed potential issues related to the randomization process (D1), deviations from the intended interventions (D2), and missing outcome data (D3). Studies classified as “high risk” were predominantly associated with missing outcome data (D3). In contrast, most trials demonstrated a low risk of bias in the measurement of the outcome (D4) and selection of the reported result (D5). Figure 2 illustrates the distribution of bias across these five domains, further supporting the reliability and interpretability of the present findings.

### 3.4 Synthesis of results

#### 3.4.1 Global cognitive function

**Figure 3:**
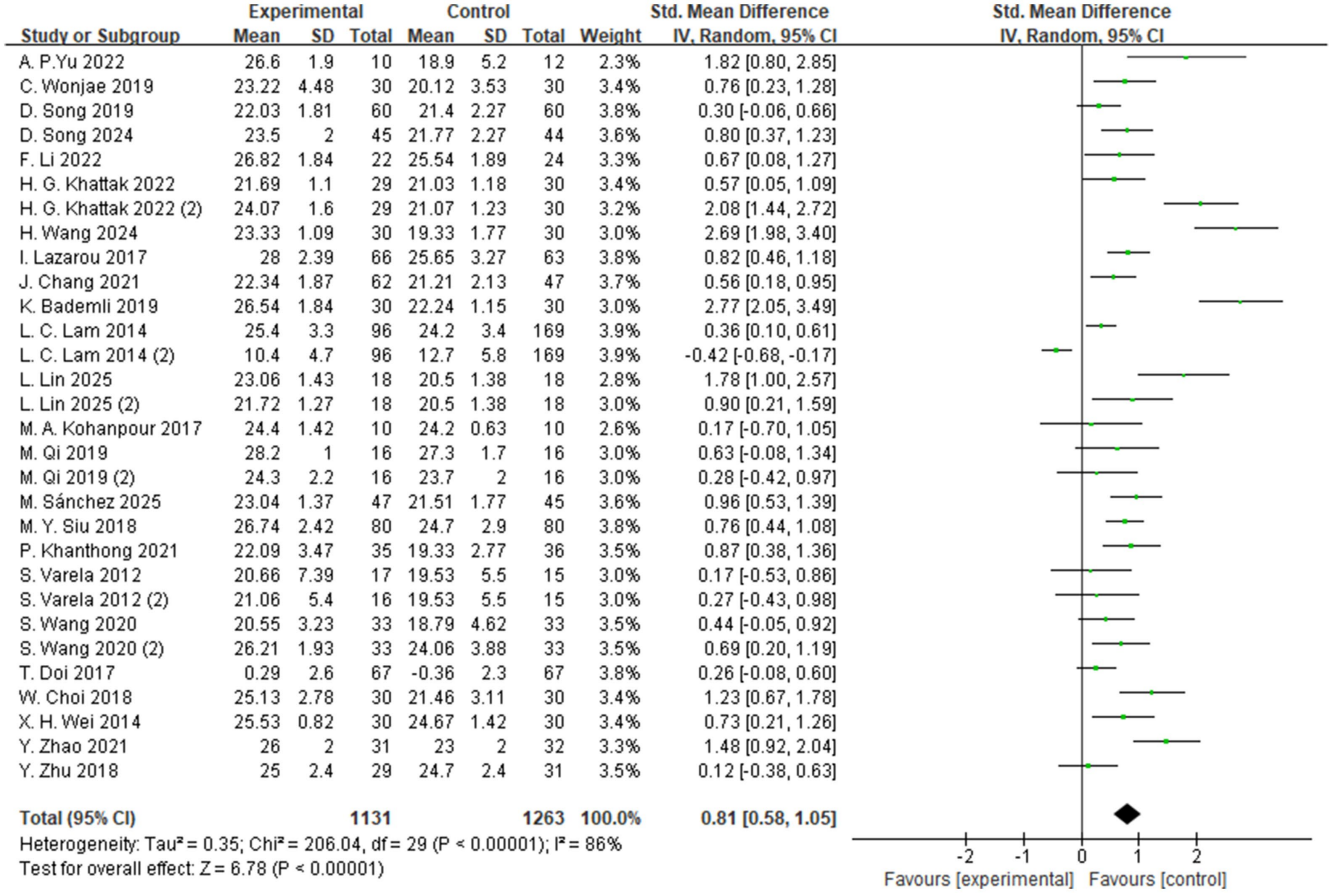
The effect of aerobic exercise on global cognitive function.

A total of 24 studies reported the effects of aerobic exercise on global cognitive function in individuals with MCI (Figure 3). Given the substantial heterogeneity among the included studies (*I*^2^ = 87%, *p* < 0.00001), a random-effects model was applied for the meta-analysis. The results indicated a statistically significant improvement in global cognitive performance in the intervention group compared to the control group (SMD = 0.81, 95% CI: 0.58 to 1.05, *Z* = 6.78, *p* < 0.00001).

To explore potential sources of heterogeneity, subgroup analyses were conducted based on variables such as intervention type, duration, intensity, frequency, and cognitive assessment tools (Appendix B). However, none of these factors sufficiently explained the observed heterogeneity. This may be attributed to variations across studies in intervention design, participant characteristics, measurement instruments, and implementation procedures. Although the primary source of heterogeneity could not be clearly identified, contextual factors such as differences in study settings, assessor training, and testing environments may have contributed to the inconsistencies in outcomes.

##### 3.4.1.1 Subgroup analyses

**Figure 4:**
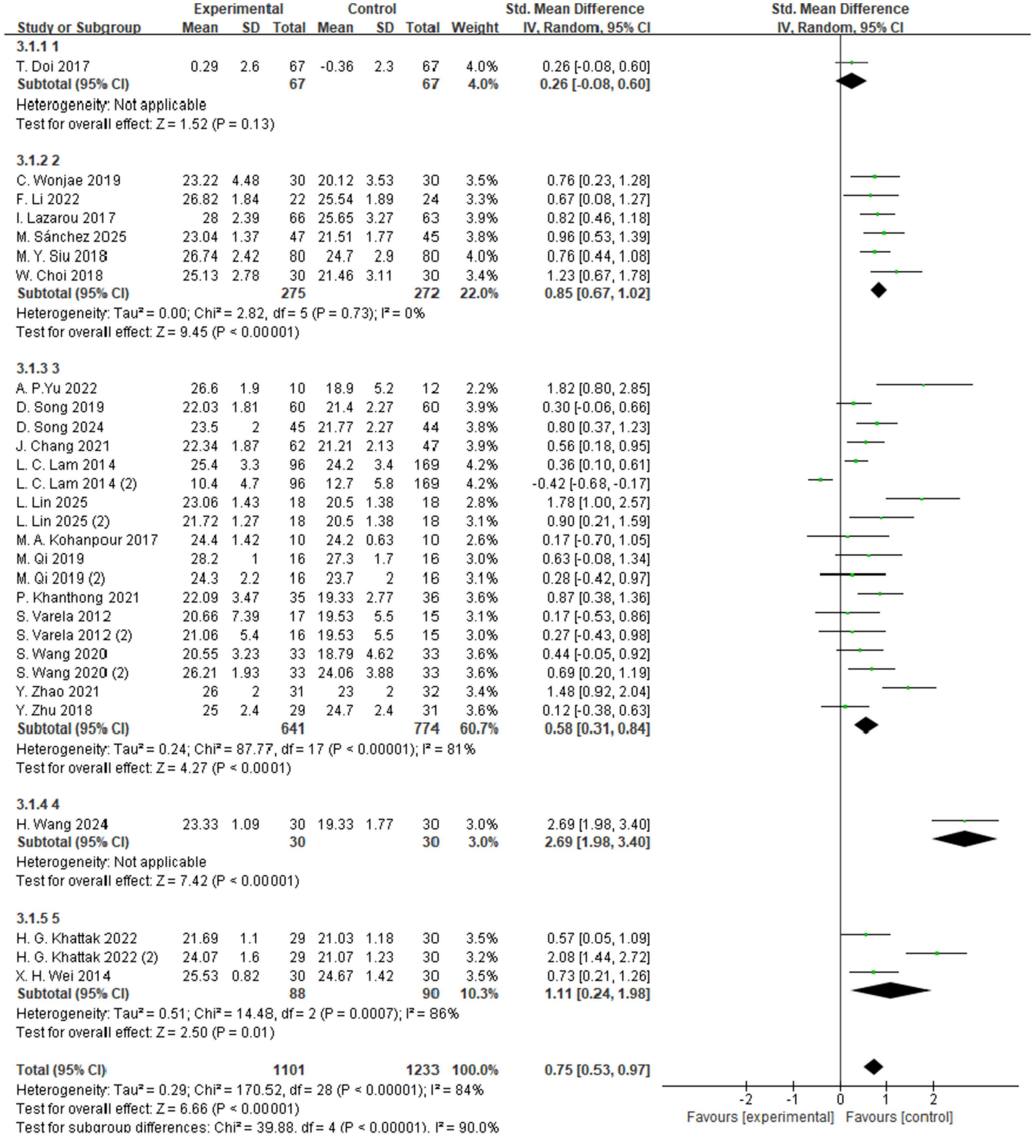
Subgroup analysis – frequency.

The subgroup analysis based on exercise frequency included studies with intervention frequencies ranging from one to five sessions per week (Figure 4). Among these, the subgroup engaging in aerobic exercise four times per week demonstrated the most significant improvement in global cognitive function, with a standardized mean difference (SMD = 2.69, 95% CI: 1.98 to 3.40, *Z* = 7.42, *p* < 0.00001). These findings suggest that a higher exercise frequency—specifically four sessions per week—may produce greater cognitive benefits for individuals with MCI.

**Figure 5:**
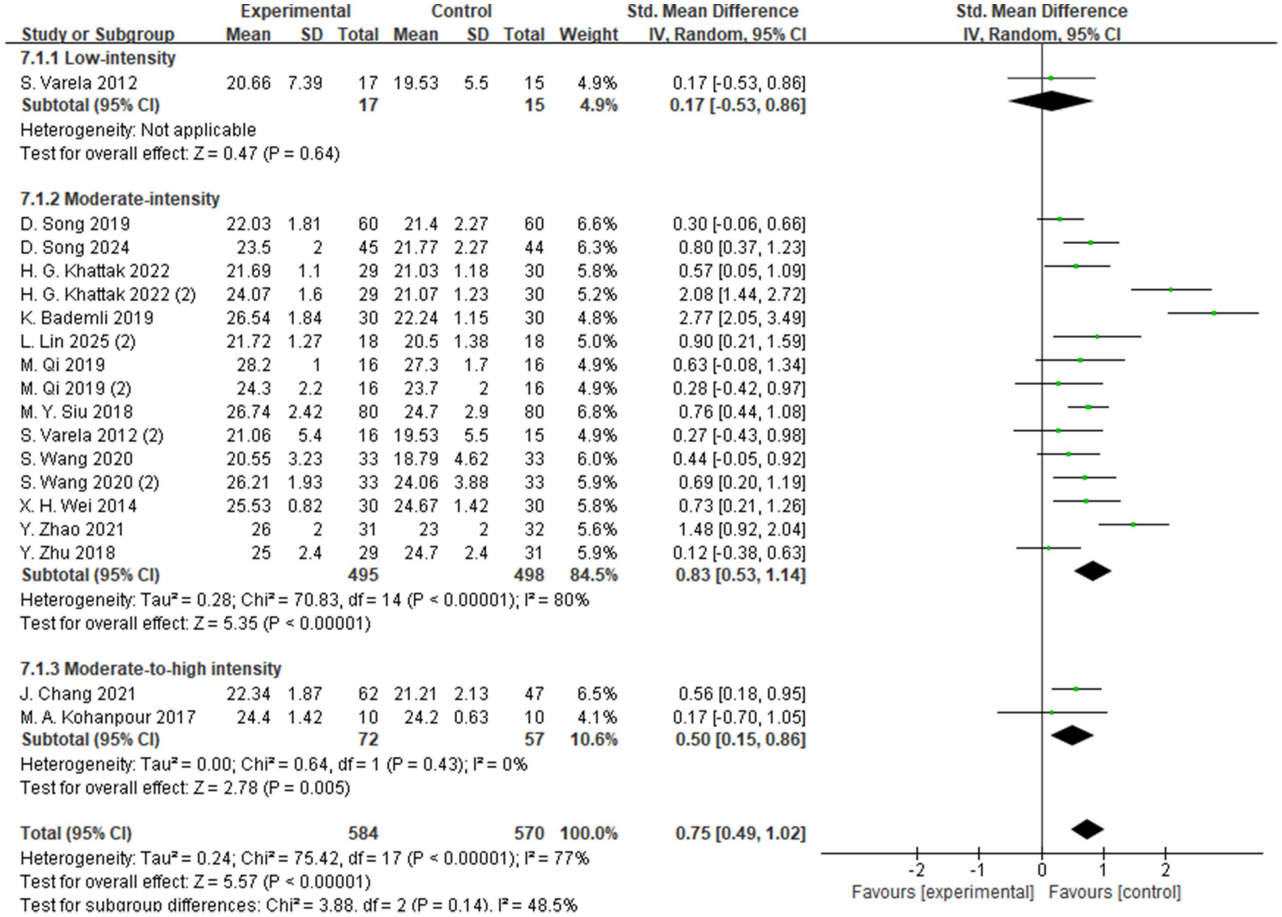
Subgroup analysis – intensity.

The subgroup analysis based on exercise intensity included studies categorized into high-, moderate-, and low-intensity intervention groups (Figure 5). The findings indicated that moderate-intensity exercise yielded the most significant improvement in global cognitive function among individuals with MCI, with a standardized mean difference (SMD = 0.83, 95% CI: 0.53 to 1.14, *Z* = 5.35, *p* < 0.00001). These results suggest that, compared to low- or high-intensity interventions, moderate-intensity aerobic exercise may be more effective in enhancing cognitive function in individuals with MCI.

**Figure 6:**
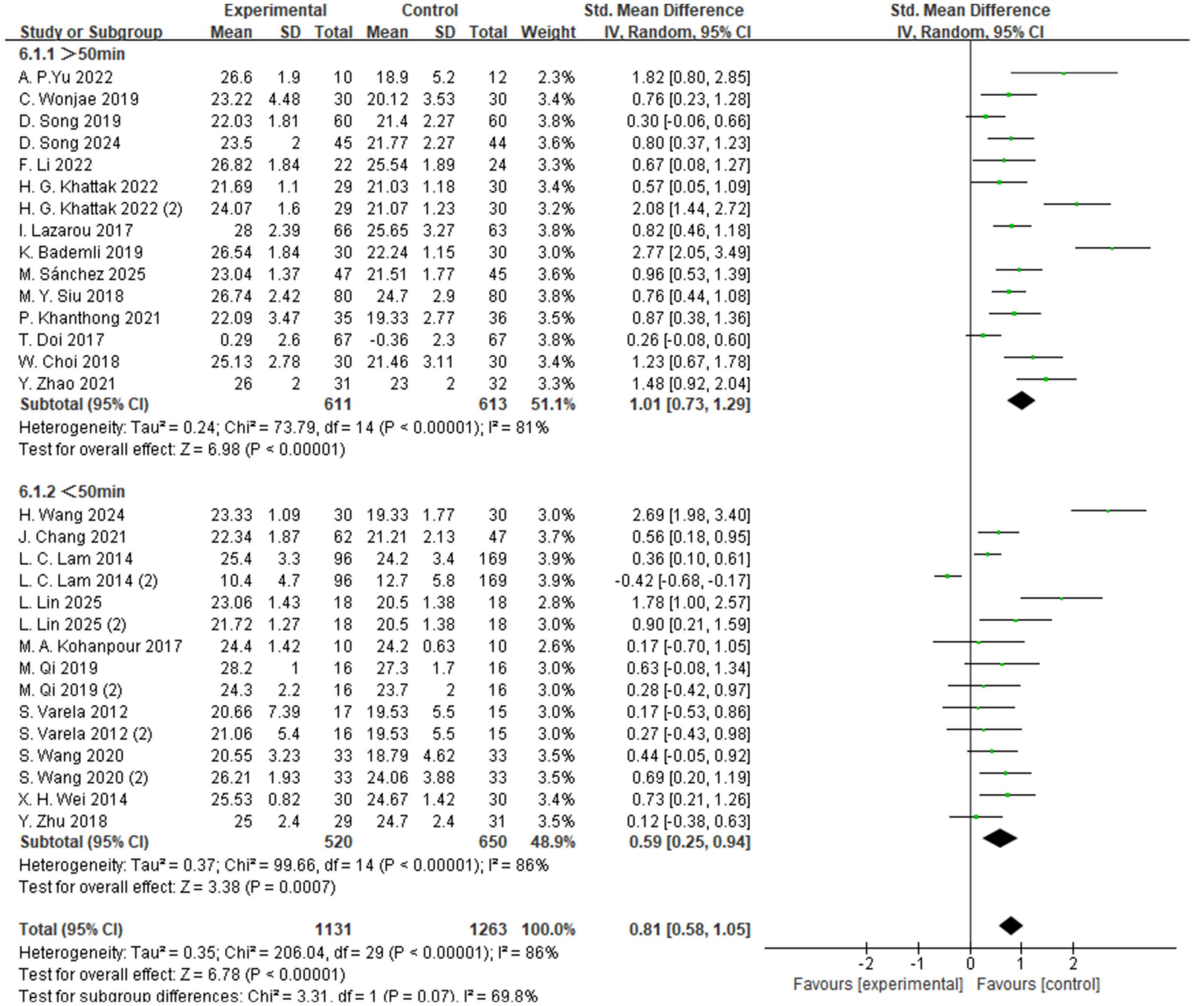
Subgroup analysis – time.

The subgroup analysis based on exercise duration categorized the interventions into two groups: sessions lasting more than 50 min and those lasting less than 50 min (Figure 6). The results showed that exercise sessions longer than 50 min produced the most significant improvement in global cognitive function among individuals with MCI, with a standardized mean difference (SMD = 1.01, 95% CI: 0.73 to 1.29, *Z* = 6.98, *p* < 0.00001). These findings suggest that, compared to shorter sessions, longer-duration aerobic exercise may be more effective in enhancing cognitive function in individuals with MCI.

**Figure 7:**
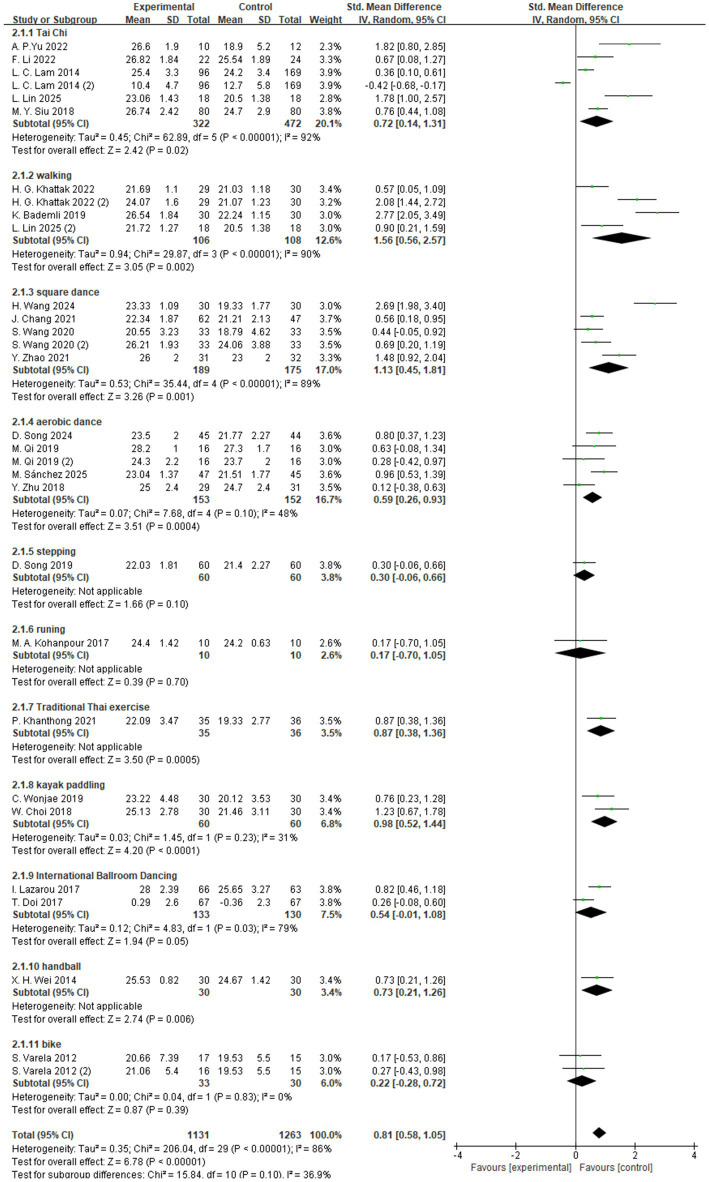
Subgroup analysis – type.

The subgroup analysis based on exercise type categorized the interventions into 11 different forms of aerobic exercise, including Tai Chi, walking, and dance (Figure 7). Among these, walking was associated with the most significant improvement in global cognitive function, with a standardized mean difference (SMD = 1.56, 95% CI: 0.56 to 2.57, *Z* = 3.05, *p* = 0.002). These findings suggest that, compared to other forms of aerobic exercise, walking may offer greater potential benefits for enhancing cognitive function in individuals with MCI.

To better interpret these results, it is important to note that the walking interventions included across studies were generally designed as moderate-intensity aerobic programs performed three to five times per week over a period of 6 to 20 weeks. Each session typically lasted 30–50 min, consisting of a warm-up, steady walking phase, and cool-down period. Exercise intensity was maintained at approximately 55–65% of the maximal heart rate or a rating of perceived exertion (RPE) of 11–14, ensuring suitability for older adults with mild cognitive impairment.

The intervention formats included both supervised treadmill or group-based training led by researchers or therapists and individualized home-based programs monitored through weekly check-ins. The methodological consistency across these studies suggests that structured, moderate-intensity, and progressively supervised walking programs may provide greater potential benefits for improving cognitive function in individuals with MCI compared with other forms of aerobic exercise.

##### 3.4.1.2 Sensitivity analysis

**Figure 8:**
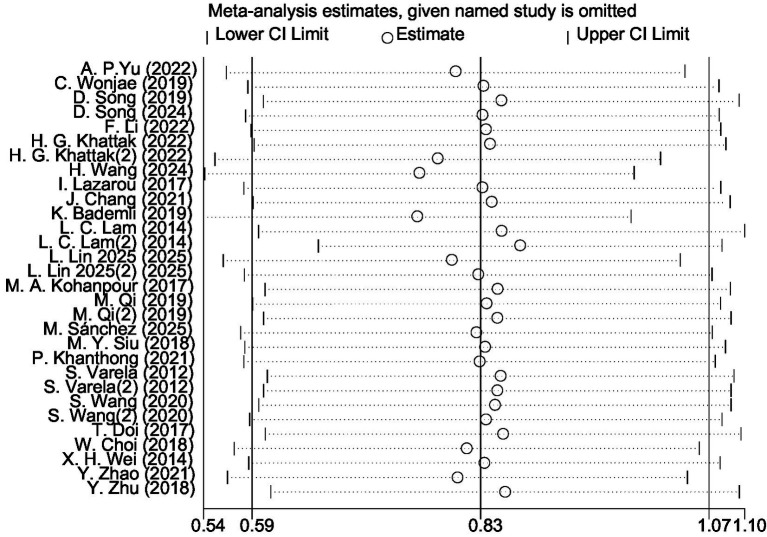
Leave-one-out sensitivity analysis plot.

The leave-one-out sensitivity analysis revealed that the magnitude and direction of the pooled effect size remained largely unchanged after sequentially excluding each study and fell within the confidence interval of the overall analysis (Figure 8). This indicates that aerobic exercise has a significant and stable effect on improving global cognitive function in patients with MCI, with the results not being substantially influenced by any single study. However, considerable heterogeneity was observed among the included studies (*I*^2^ = 87%). Exploratory inspection of study-level characteristics suggested that variations in participants’ mean age, intervention duration, and study region might partially account for this variability.

##### 3.4.1.3 Publication bias

**Figure 9:**
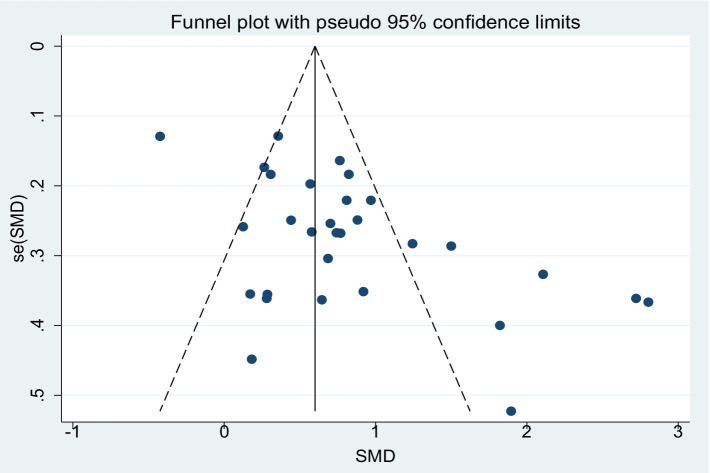
Funnel plot (Egger’s *p* = 0.001).

Figure 9 presents the funnel plot illustrating the effect of aerobic exercise on global cognitive function in patients with MCI, the distribution of which suggests the potential presence of publication bias. The Egger’s regression test further indicated significant asymmetry in the funnel plot (*p* = 0.001). To further assess the potential impact of publication bias on the results, the trim-and-fill method was applied under a random-effects model (Appendix 3). The analysis showed that no studies were imputed, and the adjusted pooled effect size (SMD = 0.827, 95% CI: 0.588–1.065, *p* < 0.001) remained consistent with the pre-adjustment estimate. These findings suggest that, despite the asymmetry observed in the funnel plot, the overall effect estimate remains robust.

#### 3.4.2 Sleep quality

**Figure 10:**
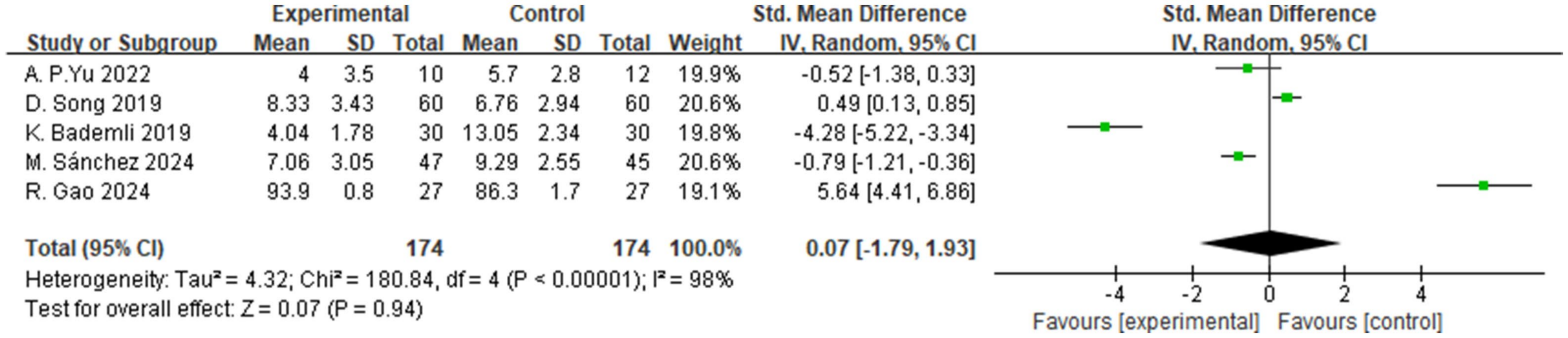
The effect of aerobic exercise on sleep quality.

A total of 5 studies assessed the effects of aerobic exercise interventions on sleep quality in individuals with MCI. As shown in Figure 10, the results indicated no statistically significant difference in sleep quality between the intervention and control groups (SMD = 0.07, 95% CI: −1.79 to 1.93, *Z* = 0.07, *p* = 0.94).

#### 3.4.3 Quality of life

**Figure 11:**
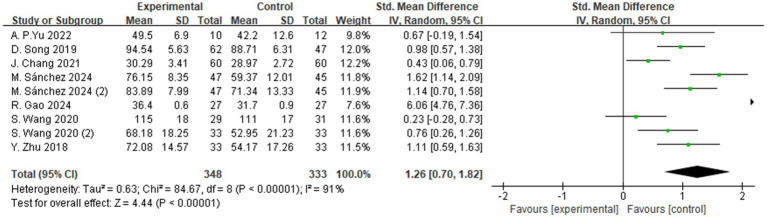
The effect of aerobic exercise on quality of life.

A total of 7 studies evaluated the effects of aerobic exercise interventions on quality of life in individuals with MCI. As shown in Figure 11, the results indicated a statistically significant improvement in quality of life in the intervention group compared to the control group (SMD = 1.26, 95% CI: 0.70 to 1.82, *Z* = 4.44, *p* < 0.00001). These findings suggest that aerobic exercise may exert a positive effect on quality of life in individuals with MCI and holds promise for clinical application.

## 4 Discussion

### 4.1 Summary of findings

This systematic review and meta-analysis synthesized data from 26 published RCTs to evaluate the effects of aerobic exercise interventions on three key domains in individuals with MCI: global cognitive function, sleep quality, and quality of life. The findings indicate that aerobic exercise has a statistically significant positive effect on enhancing global cognitive function and improving quality of life among individuals with MCI. However, no significant improvement was observed in sleep quality.

Notably, all outcome measures demonstrated substantial heterogeneity. Although the primary sources of heterogeneity could not be definitively identified, potential contributing factors include differences in participant characteristics (e.g., age, baseline cognitive status), variability in intervention designs (e.g., exercise intensity, duration, and frequency), and the diverse measurement tools employed across studies. These factors may account for the inconsistencies observed in the pooled results. In addition, the sensitivity analysis showed that, after sequentially excluding any single study, the pooled effect sizes for overall cognitive function and quality of life remained statistically significant, with only minor variations in magnitude. This suggests that the conclusions of the present study demonstrate a relatively high degree of robustness.

To further elucidate the differential effects of aerobic exercise interventions across various contexts, this study first focused on the core outcome of global cognitive function. Improvement in cognitive function is considered a primary indicator for evaluating the effectiveness of cognitive interventions, as it reflects the integrated performance of multiple cognitive domains, including memory and executive function (76). The results of this meta-analysis demonstrated that aerobic exercise significantly enhanced global cognitive function in individuals with MCI (SMD = 0.81, *p* < 0.00001). These improvements are closely associated with increased cognitive reserve, enlargement of hippocampal volume, and enhanced neuroplasticity—key factors underpinning memory, learning, and executive control (28, 30–32). Moreover, the enhancement of global cognitive function may indirectly contribute to greater independence in daily living and higher levels of social engagement, ultimately facilitating improvements in quality of life (77).

Given the significant improvements and observed heterogeneity in global cognitive outcomes, this study further conducted subgroup analyses based on the FITT framework (Frequency, Intensity, Time, and Type) to identify potential moderating factors. The findings revealed that interventions delivered four times per week produced the greatest improvements in cognitive function (SMD = 2.69, *p* < 0.00001), suggesting that regular and relatively high-frequency exercise may help maximize cognitive benefits. In contrast, lower or excessively high frequencies may lead to suboptimal outcomes due to insufficient stimulation or fatigue-related effects.

Regarding intensity, moderate-intensity aerobic exercise was associated with the most pronounced improvements in cognitive performance (SMD = 0.83, *p* < 0.00001), supporting the notion that moderate levels of stimulation may optimally activate neuroplastic mechanisms and promote cognitive enhancement (55, 57, 60). In terms of session duration, interventions lasting longer than 50 min per session yielded significantly greater cognitive gains (SMD = 1.01, *p* < 0.00001), potentially due to more robust activation of the prefrontal cortex and hippocampus during prolonged physical activity.

Finally, when examining the type of intervention, 11 distinct forms of aerobic exercise were included, such as Tai Chi, walking, and dance. Among these, walking demonstrated the greatest cognitive benefit (SMD = 1.56, *p* = 0.002), highlighting the practical value of low-barrier, easily disseminated exercise modalities for older adults with MCI.

These findings offer empirical support for the development of more targeted and effective exercise intervention strategies. By optimizing FITT parameters, it may be possible to further enhance the cognitive benefits of aerobic exercise while improving adherence and engagement among individuals with MCI.

In addition to improvements in cognitive function, this study also systematically evaluated another critical health outcome—quality of life. As the ultimate goal of cognitive interventions, enhancing quality of life represents a meaningful endpoint for individuals with MCI. The present findings demonstrate that aerobic exercise significantly improves quality of life (SMD = 1.26, *p* < 0.00001), potentially mediated through enhanced cognitive functioning. For example, interventions involving walking and dance not only improved physical health but also fostered greater social interaction and psychological well-being (57, 59, 75). Furthermore, improvements in cognitive domains—particularly memory and executive function—may enhance individuals’ self-perceived health status, suggesting a bidirectional relationship between cognitive enhancement and quality of life.

Although the observed effect size (SMD = 1.26) falls within the “large effect” range according to Cohen’s criteria, it should be interpreted with caution. Variations in measurement tools (e.g., SF-36, QOL-AD), intervention duration, and participants’ baseline health status may have influenced the magnitude of the effect. Nevertheless, this finding suggests that aerobic exercise may have clinically meaningful benefits in enhancing physical well-being among individuals with MCI. This aligns with the recent findings of Mohammadi et al. (78), who reported that structured aerobic training significantly improved quality-of-life indices, further supporting the important role of aerobic exercise in promoting overall mental and physical health (78).

Despite the non-significant findings regarding improvements in sleep quality, this outcome remains a critical area of interest due to its close association with cognitive function. Sleep disturbances are highly prevalent among individuals with MCI and are recognized as potential risk factors for cognitive decline, with proposed mechanisms involving impaired glymphatic clearance, dysregulation of prefrontal control, and alterations in emotional regulation networks (29, 53, 62, 66).

In this study, the absence of a statistically significant effect of aerobic exercise on sleep quality may be attributed to several contributing factors. First, there was heterogeneity in sleep assessment methods; some studies employed subjective instruments such as the Pittsburgh Sleep Quality Index (PSQI), while others used objective measures such as actigraphy, resulting in varying levels of sensitivity for detecting changes in sleep patterns. Second, the limited number of included trials may have reduced the overall statistical power, thereby diminishing the likelihood of detecting clinically meaningful differences. Third, variability in intervention protocols, including differences in exercise type, intensity, and duration, may also have influenced the consistency of the observed outcomes. Nevertheless, existing literature suggests that sleep quality may act as a mediator or moderator between cognitive function and quality of life (79). Future research should explore the potential of integrated intervention strategies—such as combining aerobic exercise with sleep hygiene education or cognitive behavioral therapy—to more effectively improve sleep outcomes in this population.

In summary, the findings of this study underscore the pivotal role of aerobic exercise in promoting cognitive function, sleep quality, and quality of life among individuals with MCI. Aerobic exercise may exert its beneficial effects indirectly through multiple physiological and psychosocial mechanisms suggested in previous studies, which collectively could contribute to improvements in global cognitive function and quality of life. Although the evidence for its impact on sleep quality remains inconclusive, incorporating sleep as a variable within the framework of cognitive intervention evaluation holds both theoretical and clinical relevance. Future research should further clarify the relationship between different types of exercise interventions and sleep outcomes, thereby optimizing intervention strategies. These efforts will support the development of more comprehensive and targeted exercise prescriptions for individuals with MCI.

### 4.2 Strengths and limitations

This study conducted a systematic and comprehensive analysis of global cognitive function, sleep quality, and quality of life, and proposed optimized exercise parameters based on the FITT principle to improve cognitive function in individuals with MCI, offering high clinical reference value. One of the key strengths of this study lies in its rigorous subgroup analyses, which provide a scientific basis for developing personalized exercise intervention strategies. In addition, compared to previous systematic reviews and meta-analyses on similar topics, this study included a larger number of RCTs with broader sample coverage, enhancing the representativeness and objectivity of the findings. These advantages contribute to the robustness and external validity of the conclusions and offer important insights for future clinical applications and academic research.

However, this study also has certain limitations. First, some of the included trials had relatively small sample sizes and did not provide detailed reports on participants’ adherence to the interventions or the specific intensity of the exercise protocols, which may affect the reliability of the findings. In addition, only studies published in English were included, which may have led to the omission of potentially relevant research published in other languages.

## 5 Conclusion

The findings of this study indicate that aerobic exercise has a statistically significant positive effect on enhancing global cognitive function and quality of life in older adults with MCI, while no significant improvements were observed in sleep quality.

Subgroup analyses revealed that, among older adults with MCI, interventions conducted four times per week, lasting at least 50 min per session, at moderate intensity, and primarily involving walking yielded the most substantial improvements in cognitive function. However, as the underlying mechanisms of these interventions remain unclear, further high-quality, large-scale randomized controlled trials are needed to refine and optimize exercise-based intervention strategies.

## Data Availability

The original contributions presented in the study are included in the article/Supplementary material, further inquiries can be directed to the corresponding author.

## References

[1] Daffner KR. Promoting successful cognitive aging: a comprehensive review. J Alzheimer's Dis. (2010) 19:1101–22. doi: 10.3233/JAD-2010-1306, 20308777 PMC3047597

[2] World Health Organization. Risk reduction of cognitive decline and dementia: WHO guidelines. Geneva: World Health Organization (2019). 31219687

[3] Giebel CM Sutcliffe C Challis D. Activities of daily living and quality of life across different stages of dementia: a UK study. Aging Ment Health. (2015) 19:63–71. doi: 10.1080/13607863.2014.915920, 24831511

[4] Han C Sun W Zhang D Xi X Zhang R Gong W. Effects of different aerobic exercises on the global cognitive function of the elderly with mild cognitive impairment: a meta-analysis. BMJ Open. (2023) 13:e067293. doi: 10.1136/bmjopen-2022-067293, 37399446 PMC10314475

[5] Jongsiriyanyong S Limpawattana P. Mild cognitive impairment in clinical practice: a review article. Am J Alzheimers Dis Other Dement. (2018) 33:500–7. doi: 10.1177/1533317518791401, 30068225 PMC10852498

[6] Petersen RC Smith GE Waring SC Ivnik RJ Tangalos EG Kokmen E. Mild cognitive impairment: clinical characterization and outcome. Arch Neurol. (1999) 56:303–8. doi: 10.1001/archneur.56.3.303, 10190820

[7] Trzepacz PT Hochstetler H Wang S Walker B Saykin AJ Alzheimer’s Disease Neuroimaging Initiative. Relationship between the Montreal cognitive assessment and mini-mental state examination for assessment of mild cognitive impairment in older adults. BMC Geriatr. (2015) 15:107. doi: 10.1186/s12877-015-0103-3 26346644 PMC4562190

[8] Hao L Wang X Zhang L Xing Y Guo Q Hu X . Prevalence, risk factors, and complaints screening tool exploration of subjective cognitive decline in a large cohort of the Chinese population. J Alzheimer's Dis. (2017) 60:371–88. doi: 10.3233/JAD-170347, 28869471

[9] McGrattan AM Pakpahan E Siervo M Mohan D Reidpath DD Prina M . Risk of conversion from mild cognitive impairment to dementia in low- and middle-income countries: a systematic review and meta-analysis. Alzheimers Dement. (2022) 8:e12267. doi: 10.1002/trc2.12267, 35310524 PMC8918697

[10] Tschanz JT Welsh-Bohmer KA Lyketsos CG Corcoran C Green RC Hayden K . Conversion to dementia from mild cognitive disorder: the Cache County study. Neurology. (2006) 67:229–34. doi: 10.1212/01.wnl.0000224748.48011.84, 16864813

[11] Winblad B Palmer K Kivipelto M Jelic V Fratiglioni L Wahlund LO . Mild cognitive impairment – beyond controversies, towards a consensus: report of the international working group on mild cognitive impairment. J Intern Med. (2004) 256:240–6. doi: 10.1111/j.1365-2796.2004.01380.x, 15324367

[12] Cooper C Li R Lyketsos C Livingston G. Treatment for mild cognitive impairment: systematic review. Br J Psychiatry. (2013) 203:255–64. doi: 10.1192/bjp.204.1.81 24085737 PMC3943830

[13] Fink HA Jutkowitz E McCarten JR Hemmy LS Butler M Davila H . Pharmacologic interventions to prevent cognitive decline, mild cognitive impairment, and clinical Alzheimer-type dementia: a systematic review. Ann Intern Med. (2018) 168:39–351. doi: 10.7326/M17-1529 29255847

[14] Iadecola C Yaffe K Biller J Bratzke LC Faraci FM Gorelick PB . Impact of hypertension on cognitive function: a scientific statement from the American Heart Association. Hypertension. (2016) 68:e67–94. doi: 10.1161/HYP.0000000000000053, 27977393 PMC5361411

[15] Wang J Tan L Wang HF Tan CC Meng XF Wang C . Anti-inflammatory drugs and risk of Alzheimer's disease: an updated systematic review and meta-analysis. J Alzheimer's Dis. (2015) 44:385–96. doi: 10.3233/JAD-141506, 25227314

[16] Arcoverde C Deslandes A Moraes H Almeida C Araujo NBD Vasques PE . Treadmill training as an augmentation treatment for Alzheimer’s disease: a pilot randomized controlled study. Arq Neuropsiquiatr. (2014) 72:190–6. doi: 10.1590/0004-282X20130231, 24676435

[17] Barban F Annicchiarico R Pantelopoulos S Federici A Perri R Fadda L . Protecting cognition from aging and Alzheimer's disease: a computerized cognitive training combined with reminiscence therapy. Int J Geriatr Psychiatry. (2016) 31:340–8. doi: 10.1002/gps.4328, 26205305

[18] Gallego MG García JG. Music therapy and Alzheimer's disease: cognitive, psychological, and behavioural effects. Neurologia. (2017) 32:300–8. doi: 10.1016/j.nrleng.2015.12.001 26896913

[19] Hoffmann K Sobol NA Frederiksen KS Beyer N Vogel A Vestergaard K . Moderate-to-high intensity physical exercise in patients with Alzheimer’s disease: a randomized controlled trial. J Alzheimer's Dis. (2016) 50:443–53. doi: 10.3233/JAD-150817, 26682695

[20] Li CH Liu CK Yang YH Chou MC Chen CH Lai CL. Adjunct effect of music therapy on cognition in Alzheimer’s disease in Taiwan: a pilot study. Neuropsychiatr Dis Treat. (2015) 11:291–6. doi: 10.2147/NDT.S73928, 25678794 PMC4322884

[21] Tárraga L Boada M Modinos G Espinosa A Diego S Morera A . A randomized pilot study to assess the efficacy of an interactive, multimedia tool of cognitive stimulation in Alzheimer’s disease. J Neurol Neurosurg Psychiatry. (2006) 77:1116–21. doi: 10.1136/jnnp.2005.086074 16820420 PMC2077529

[22] Venturelli M Scarsini R Schena F. Six-month walking program changes cognitive and ADL performance in patients with Alzheimer. Am J Alzheimers Dis Other Dement. (2011) 26:381–8. doi: 10.1177/1533317511418956, 21852281 PMC10845333

[23] Dasso NA. How is exercise different from physical activity? A concept analysis. Nurs Forum. (2019) 54:45–52. doi: 10.1111/nuf.12296 30332516

[24] Teixeira CVL Gobbi LTB Corazza DI Stella F Costa JLR Gobbi S. Non-pharmacological interventions on cognitive functions in older people with mild cognitive impairment (MCI). Arch Gerontol Geriatr. (2012) 54:175–80. doi: 10.1016/j.archger.2011.02.014, 21397960

[25] Gallaway PJ Miyake H Buchowski MS Shimada M Yoshitake Y Kim AS . Physical activity: a viable way to reduce the risks of mild cognitive impairment, Alzheimer’s disease, and vascular dementia in older adults. Brain Sci. (2017) 7:22. doi: 10.3390/brainsci7020022, 28230730 PMC5332965

[26] American College of Sports Medicine. ACSM'S guidelines for exercise testing and prescription. 4th ed. Philadelphia: Lippincott Williams & Wilkins (2013). 10.1249/JSR.0b013e31829a68cf 23851406

[27] Song D Doris SF Li PW Lei Y. The effectiveness of physical exercise on cognitive and psychological outcomes in individuals with mild cognitive impairment: a systematic review and meta-analysis. Int J Nurs Stud. (2018) 79:155–64. doi: 10.1016/j.ijnurstu.2018.01.002 29334638

[28] Heijnen S Hommel B Kibele A Colzato LS. Neuromodulation of aerobic exercise—a review. Front Psychol. (2016) 6:1890. doi: 10.3389/fpsyg.2015.01890, 26779053 PMC4703784

[29] Song D Doris SF. Effects of a moderate-intensity aerobic exercise programme on the cognitive function and quality of life of community-dwelling elderly people with mild cognitive impairment: a randomised controlled trial. Int J Nurs Stud. (2019) 93:97–105. doi: 10.1016/j.ijnurstu.2019.02.019 30901716

[30] Tsai CL Ukropec J Ukropcová B Pai MC. An acute bout of aerobic or strength exercise specifically modifies circulating exerkine levels and neurocognitive functions in elderly individuals with mild cognitive impairment. NeuroImage Clin. (2018) 17:272–84. doi: 10.1016/j.nicl.2017.10.028, 29527475 PMC5842646

[31] Curlik DM Shors TJ. Training your brain: do mental and physical (MAP) training enhance cognition through the process of neurogenesis in the hippocampus? Neuropharmacology. (2013) 64:506–14. doi: 10.1016/j.neuropharm.2012.07.027, 22898496 PMC3445739

[32] Kempermann G Fabel K Ehninger D Babu H Leal-Galicia P Garthe A . Why and how physical activity promotes experience-induced brain plasticity. Front Neurosci. (2010) 4:189. doi: 10.3389/fnins.2010.00189, 21151782 PMC3000002

[33] Rehfeld K Lüders A Hökelmann A Lessmann V Kaufmann J Brigadski T . Dance training is superior to repetitive physical exercise in inducing brain plasticity in the elderly. PLoS One. (2018) 13:e0196636. doi: 10.1371/journal.pone.0196636, 29995884 PMC6040685

[34] Vidoni ED Van Sciver A Johnson DK He J Honea R Haines B . A community-based approach to trials of aerobic exercise in aging and Alzheimer's disease. Contemp Clin Trials. (2012) 33:1105–16. doi: 10.1016/j.cct.2012.08.002, 22903151 PMC3468654

[35] McKinnon A Terpening Z Hickie IB Batchelor J Grunstein R Lewis SJ . Prevalence and predictors of poor sleep quality in mild cognitive impairment. J Geriatr Psychiatry Neurol. (2014) 27:204–11. doi: 10.1177/0891988714527516, 24687189

[36] Smith L Shin JI Jacob L Carmichael C Sánchez GFL Oh H . Sleep problems and mild cognitive impairment among adults aged ≥50 years from low- and middle-income countries. Exp Gerontol. (2021) 154:111513. doi: 10.1016/j.exger.2021.111513 34384889

[37] Song D Yu DS Sun Q He G. Correlates of sleep disturbance among older adults with mild cognitive impairment: a cross-sectional study. Int J Environ Res Public Health. (2020) 17:4862. doi: 10.3390/ijerph17134862, 32640633 PMC7369813

[38] Alperin N Wiltshire J Lee SH Ramos AR Hernandez-Cardenache R Rundek T . Effect of sleep quality on amnestic mild cognitive impairment vulnerable brain regions in cognitively normal elderly individuals. Sleep. (2019) 42:zsy254. doi: 10.1093/sleep/zsy254, 30541112 PMC6424074

[39] Palmer K Mitolo M Burgio F Meneghello F Venneri A. Sleep disturbance in mild cognitive impairment and association with cognitive functioning: a case-control study. Front Aging Neurosci. (2018) 10:360. doi: 10.3389/fnagi.2018.00360, 30473661 PMC6237832

[40] Bárrios H Narciso S Guerreiro M Maroco J Logsdon R de Mendonça A. Quality of life in patients with mild cognitive impairment. Aging Ment Health. (2013) 17:287–92. doi: 10.1080/13607863.2012.747083, 23215827

[41] Missotten P Squelard G Ylieff M Di Notte D Paquay L De Lepeleire J . Quality of life in older Belgian people: comparison between people with dementia, mild cognitive impairment, and controls. Int J Geriatr Psychiatry. (2008) 23:1103–9. doi: 10.1002/gps.1981, 18213606

[42] Ready RE Ott BR Grace J. Patient versus informant perspectives of quality of life in mild cognitive impairment and Alzheimer's disease. Int J Geriatr Psychiatry. (2004) 19:256–65. doi: 10.1002/gps.1075, 15027041

[43] Logsdon RG Gibbons LE McCurry SM Teri L. Assessing quality of life in older adults with cognitive impairment. Psychosom Med. (2002) 64:510–9. doi: 10.1097/00006842-200205000-00016, 12021425

[44] Ahn J Kim M. Effects of aerobic exercise on global cognitive function and sleep in older adults with mild cognitive impairment: a systematic review and meta-analysis. Geriatr Nurs. (2023) 51:9–16. doi: 10.1016/j.gerinurse.2023.02.008, 36871328

[45] Huang CS Yan YJ Luo YT Lin R Li H. Effects of dance therapy on cognitive and mental health in adults aged 55 years and older with mild cognitive impairment: a systematic review and meta-analysis. BMC Geriatr. (2023) 23:695. doi: 10.1186/s12877-023-04406-y, 37880590 PMC10601250

[46] Liu X Wang G Cao Y. The effectiveness of exercise on global cognitive function, balance, depression symptoms, and sleep quality in patients with mild cognitive impairment: a systematic review and meta-analysis. Geriatr Nurs. (2023) 51:182–93. doi: 10.1016/j.gerinurse.2023.03.013, 37011490

[47] Lee J. Effects of aerobic and resistance exercise interventions on cognitive and physiologic adaptations for older adults with mild cognitive impairment: a systematic review and meta-analysis of randomized control trials. Int J Environ Res Public Health. (2020) 17:9216. doi: 10.3390/ijerph17249216, 33317169 PMC7764103

[48] Cumpston M Li T Page MJ Chandler J Welch VA Higgins JP . Updated guidance for trusted systematic reviews: a new edition of the Cochrane handbook for systematic reviews of interventions. Cochrane Database Syst Rev. (2019) 10:ED000142. doi: 10.1002/14651858.ED000142, 31643080 PMC10284251

[49] Deeks JJ Higgins JP Altman DG Cochrane Statistical Methods Group. Analysing data and undertaking meta-analyses In: Higgins JP Thomas J Chandler J Cumpston M Li T Page MJ , editors. Cochrane handbook for systematic reviews of interventions. 2nd ed. Chichester: Wiley (2019). 241–84.

[50] Cohen J. Statistical power analysis for the behavioral sciences. 2nd ed. New York: Academic Press (2013).

[51] Huedo-Medina TB Sánchez-Meca J Marín-Martínez F Botella J. Assessing heterogeneity in meta-analysis: Q statistic or I^2^ index? Psychol Methods. (2006) 11:193–206. doi: 10.1037/1082-989X.11.2.193, 16784338

[52] Chang J Zhu W Zhang J Yong L Yang M Wang J . The effect of Chinese square dance exercise on cognitive function in older women with mild cognitive impairment: the mediating effect of mood status and quality of life. Front Psych. (2021) 12:711079. doi: 10.3389/fpsyt.2021.711079, 34305689 PMC8298898

[53] Gao R Greiner C Ryuno H Zhang X. Effects of tai chi on physical performance, sleep, and quality of life in older adults with mild to moderate cognitive impairment. BMC Complement Med Ther. (2024) 24:423. doi: 10.1186/s12906-024-04705-w, 39716129 PMC11667984

[54] Lin L He YX Wen Q Liu JY Dai Y Fei YZ . Evaluation of the efficacy of tai chi on the cognitive function of patients with mild cognitive dysfunction and research on its mechanism. Front Aging Neurosci. (2025) 17:1435996. doi: 10.3389/fnagi.2025.1435996, 40264462 PMC12012717

[55] Qi M Zhu YI Zhang L Wu T Wang JIE. The effect of aerobic dance intervention on brain spontaneous activity in older adults with mild cognitive impairment: a resting-state functional MRI study. Exp Ther Med. (2019) 17:715–22. doi: 10.3892/etm.2018.7006, 30651855 PMC6307442

[56] Song D Yu D Liu T Wang J. Effect of an aerobic dancing program on sleep quality for older adults with mild cognitive impairment and poor sleep: a randomized controlled trial. J Am Med Dir Assoc. (2024) 25:494–9. doi: 10.1016/j.jamda.2023.09.020, 39492163

[57] Wang S Yin H Meng X Shang B Meng Q Zheng L . Effects of Chinese square dancing on older adults with mild cognitive impairment. Geriatr Nurs. (2020) 41:290–6. doi: 10.1016/j.gerinurse.2019.10.009, 31727347

[58] Wei XH Ji LL. Effect of handball training on cognitive ability in elderly with mild cognitive impairment. Neurosci Lett. (2014) 566:98–101. doi: 10.1016/j.neulet.2014.02.035, 24582900

[59] Zhao Y Yin M Yao X Li Z. Effects of nurse-led square dancing on older patients with mild cognitive impairment combined with depressive symptoms: a pilot study. Geriatr Nurs. (2021) 42:1164–71. doi: 10.1016/j.gerinurse.2021.06.013 34425421

[60] Zhu Y Wu H Qi M Wang S Zhang Q Zhou L . Effects of a specially designed aerobic dance routine on mild cognitive impairment. Clin Interv Aging. (2018) 13:1691–700. doi: 10.2147/CIA.S163067, 30237705 PMC6138969

[61] Sánchez-Alcalá M Aibar-Almazán A Carcelén-Fraile MDC Castellote-Caballero Y Cano-Sánchez J Achalandabaso-Ochoa A . Effects of dance-based aerobic training on frailty and cognitive function in older adults with mild cognitive impairment: a randomized controlled trial. Diagnostics. (2025) 15:351. doi: 10.3390/diagnostics15030351, 39941281 PMC11817983

[62] Sánchez-Alcalá M Aibar-Almazán A Hita-Contreras F Castellote-Caballero Y Carcelén-Fraile MDC Infante-Guedes A . Effects of dance-based aerobic training on mental health and quality of life in older adults with mild cognitive impairment. J Pers Med. (2024) 14:844. doi: 10.3390/jpm14080844, 39202035 PMC11355123

[63] Varela S Ayán C Cancela JM Martín V. Effects of two different intensities of aerobic exercise on elderly people with mild cognitive impairment: a randomized pilot study. Clin Rehabil. (2012) 26:442–50. doi: 10.1177/0269215511425835, 22116953

[64] Lam LC Chan WM Kwok TC Chiu HF. Effectiveness of tai chi in maintenance of cognitive and functional abilities in mild cognitive impairment: a randomised controlled trial. Hong Kong Med J. (2014) 20:20–3. 25001031

[65] Siu MY Lee DT. Effects of tai chi on cognition and instrumental activities of daily living in community dwelling older people with mild cognitive impairment. BMC Geriatr. (2018) 18:37. doi: 10.1186/s12877-018-0720-8, 29394884 PMC5797349

[66] Yu AP Chin EC Yu DJ Fong DY Cheng CP Hu X . Tai chi versus conventional exercise for improving cognitive function in older adults: a pilot randomized controlled trial. Sci Rep. (2022) 12:8868. doi: 10.1038/s41598-022-12526-5 35614144 PMC9131984

[67] Choi W Lee S. Ground kayak paddling exercise improves postural balance, muscle performance, and cognitive function in older adults with mild cognitive impairment: a randomized controlled trial. Med Sci Monit. (2018) 24:3909–15. doi: 10.12659/MSM.908248, 29886507 PMC6026380

[68] Choi W Lee S. The effects of virtual kayak paddling exercise on postural balance, muscle performance, and cognitive function in older adults with mild cognitive impairment: a randomized controlled trial. J Aging Phys Act. (2019) 27:861–70. doi: 10.1123/japa.2018-0020, 31034303

[69] Li F Harmer P Fitzgerald K Winters-Stone K. A cognitively enhanced online tai Ji Quan training intervention for community-dwelling older adults with mild cognitive impairment: a feasibility trial. BMC Geriatr. (2022) 22:76. doi: 10.1186/s12877-021-02747-0, 35078407 PMC8787180

[70] Kohanpour MA Peeri M Azarbayjani MA. The effects of aerobic exercise with lavender essence use on cognitive state and serum brain-derived neurotrophic factor levels in elderly with mild cognitive impairment. J Herbmed Pharmacol. (2017) 6:80–4.

[71] Khanthong P Sriyakul K Dechakhamphu A Krajarng A Kamalashiran C Tungsukruthai P. Traditional Thai exercise (Ruesi Dadton) for improving motor and cognitive functions in mild cognitive impairment: a randomized controlled trial. J Exerc Rehabil. (2021) 17:331–8. doi: 10.12965/jer.2142542.271, 34805022 PMC8566108

[72] Doi T Verghese J Makizako H Tsutsumimoto K Hotta R Nakakubo S . Effects of cognitive leisure activity on cognition in mild cognitive impairment: results of a randomized controlled trial. J Am Med Dir Assoc. (2017) 18:686–91. doi: 10.1016/j.jamda.2017.02.013, 28396179

[73] Khattak HG Ahmad Z Arshad H Anwar K. Effect of aerobic exercise on cognition in elderly persons with mild cognitive impairment. Rawal Med J. (2022) 47:696–6. doi: 10.5455/rmj.20210713072242

[74] Lazarou I Parastatidis T Tsolaki A Gkioka M Karakostas A Douka S . International ballroom dancing against neurodegeneration: a randomized controlled trial in Greek community-dwelling elders with mild cognitive impairment. Am J Alzheimers Dis Other Dement. (2017) 32:489–99. doi: 10.1177/1533317517725813, 28840742 PMC10852896

[75] Bademli K Lok N Canbaz M Lok S. Effects of physical activity program on cognitive function and sleep quality in elderly with mild cognitive impairment: a randomized controlled trial. Perspect Psychiatr Care. (2019) 55:401–8. doi: 10.1111/ppc.12324, 30430592

[76] Liu T Wen W Zhu W Kochan NA Trollor JN Reppermund S . The relationship between cortical sulcal variability and cognitive performance in the elderly. NeuroImage. (2011) 56:865–73. doi: 10.1016/j.neuroimage.2011.03.015, 21397704

[77] Pusswald G Tropper E Kryspin-Exner I Moser D Klug S Auff E . Health-related quality of life in patients with subjective cognitive decline and mild cognitive impairment and its relation to activities of daily living. J Alzheimer's Dis. (2015) 47:479–86. doi: 10.3233/JAD-150284, 26401569

[78] Mohammadi M Eftekhari E Banaei BJ Zahedi H. Comparison of spinning and resistance training on resistin, visfatin, lipid profile, and quality of life in overweight women. Health. (2025) 3:42–53. doi: 10.61838/kman.hn.3.2.6

[79] Hatami F Atani SA Caron V Yousefi S. Exploring the effects of physical activity levels and sleep quality on cognitive failure in elderly: a cross-sectional study. Int J Sport Stud Health. (2025) 8:1–15. doi: 10.61838/kman.intjssh.8.4.6

[80] Wang H Pei Z Liu Y. Effects of square dance exercise on cognitive function in elderly individuals with mild cognitive impairment: the mediating role of balance ability and executive function. BMC Geriatr. (2024) 24:156. doi: 10.1186/s12877-024-04714-x, 38360628 PMC10870555

[81] Shu W Kim S. Effects of aerobic exercise interventions on cognitive function and quality of life in older adults with mild cognitive impairment: a systematic review and meta-analysis, (2024). [Epubh ahead of preprint]. doi: 10.21203/rs.3.rs-4455613/v1. PMC12753406 41479612

