# Supplementary material for: Effects of aerobic exercise interventions on cognitive function, sleep quality, and quality of life in older adults with mild cognitive impairment: a systematic review and meta-analysis
Source: Front Neurol. 2025 Dec 17;16:1693052. doi: 10.3389/fneur.2025.1693052 (PMC12753406; doi:10.3389/fneur.2025.1693052)
Supplement: Supplementary file 1 [file Table_1.DOCX]

**Subject Headings and Free terms**

**Participants（P）**：Mild Cognitive Impairment

# **Medical Subject Headings：**Cognitive Dysfunction

**Free terms：**Cognitive Dysfunctions

Dysfunction, Cognitive

Dysfunctions, Cognitive

Cognitive Impairments

Cognitive Impairment

Impairment, Cognitive

Impairments, Cognitive

Cognitive Disorder

Cognitive Disorders

Disorder, Cognitive

Disorders, Cognitive

Mild Cognitive Impairment

Cognitive Impairment, Mild

Cognitive Impairments, Mild

Impairment, Mild Cognitive

Impairments, Mild Cognitive

Mild Cognitive Impairments

Cognitive Decline

Cognitive Declines

Decline, Cognitive

Declines, Cognitive

Mental Deterioration

Deterioration, Mental

Deteriorations, Mental

Mental Deteriorations

**Intervention（I）：**Aerobic Exercise

# **Medical Subject Headings：**Exercise

**Free terms：**Exercises

Physical Activity

Activities, Physical

Activity, Physical

Physical Activities

Exercise, Physical

Exercises, Physical

Physical Exercise

Physical Exercises

Acute Exercise

Acute Exercises

Isometric Exercise

Exercise, Aerobic

Aerobic Exercise

Aerobic Exercises

Exercises, Aerobic

Exercise Training

Exercise Trainings

Training, Exercise

Trainings, Exercise

Yoga

Tai Chi Chuan

Running

Jogging

Walking

Dancing

Swimming

Cycling

Rope skipping

**Study Design Types（S）：**

**Search Strategy：**

randomized controlled trial[Publication Type] OR randomized[Title/Abstract] OR
placebo[Title/Abstract]

**Search Strategy**

**Pubmed Search Strategy:**

((("Cognitive Dysfunction"[Mesh]) OR (((((((((((((((((((((((((Cognitive Dysfunctions[Title/Abstract]) OR (Dysfunction, Cognitive[Title/Abstract])) OR (Dysfunctions, Cognitive[Title/Abstract])) OR (Cognitive Impairments[Title/Abstract])) OR (Cognitive Impairment[Title/Abstract])) OR (Impairment, Cognitive[Title/Abstract])) OR (Impairments, Cognitive[Title/Abstract])) OR (Cognitive Disorder[Title/Abstract])) OR (Cognitive Disorders[Title/Abstract])) OR (Disorder, Cognitive[Title/Abstract])) OR (Disorders, Cognitive[Title/Abstract])) OR (Mild Cognitive Impairment[Title/Abstract])) OR (Cognitive Impairment, Mild[Title/Abstract])) OR (Cognitive Impairments, Mild[Title/Abstract])) OR (Impairment, Mild Cognitive[Title/Abstract])) OR (Impairments, Mild Cognitive[Title/Abstract])) OR (Mild Cognitive Impairments[Title/Abstract])) OR (Cognitive Decline[Title/Abstract])) OR (Cognitive Declines[Title/Abstract])) OR (Decline, Cognitive[Title/Abstract])) OR (Declines, Cognitive[Title/Abstract])) OR (Mental Deterioration[Title/Abstract])) OR (Deterioration, Mental[Title/Abstract])) OR (Deteriorations, Mental[Title/Abstract])) OR (Mental Deteriorations[Title/Abstract]))) AND (("Exercise"[Mesh]) OR (((((((((((((((((((((((((((((Exercises[Title/Abstract]) OR (Physical Activity[Title/Abstract])) OR (Activities, Physical[Title/Abstract])) OR (Activity, Physical[Title/Abstract])) OR (Physical Activities[Title/Abstract])) OR (Exercise, Physical[Title/Abstract])) OR (Exercises, Physical[Title/Abstract])) OR (Physical Exercise[Title/Abstract])) OR (Physical Exercises[Title/Abstract])) OR (Acute Exercise[Title/Abstract])) OR (Acute Exercises[Title/Abstract])) OR (Isometric Exercise[Title/Abstract])) OR (Exercise, Aerobic[Title/Abstract])) OR (Aerobic Exercise[Title/Abstract])) OR (Aerobic Exercises[Title/Abstract])) OR (Exercises, Aerobic[Title/Abstract])) OR (Exercise Training[Title/Abstract])) OR (Exercise Trainings[Title/Abstract])) OR (Training, Exercise[Title/Abstract])) OR (Trainings, Exercise[Title/Abstract])) OR (Yoga[Title/Abstract])) OR (Tai Chi Chuan[Title/Abstract])) OR (Running[Title/Abstract])) OR (Jogging[Title/Abstract])) OR (Walking[Title/Abstract])) OR (Dancing[Title/Abstract])) OR (Swimming[Title/Abstract])) OR (Cycling[Title/Abstract])) OR (Rope skipping[Title/Abstract])))) AND (randomized controlled trial[Publication Type] OR randomized[Title/Abstract] OR placebo[Title/Abstract])

**Embase Search Strategy:**

'Cognitive Dysfunctions':ab,ti OR 'Dysfunction, Cognitive':ab,ti OR 'Dysfunctions, Cognitive':ab,ti OR 'Cognitive Impairments':ab,ti OR 'Cognitive Impairment':ab,ti OR 'Impairment, Cognitive':ab,ti OR 'Impairments, Cognitive':ab,ti OR 'Cognitive Disorder':ab,ti OR 'Cognitive Disorders':ab,ti OR 'Disorder, Cognitive':ab,ti OR 'Disorders, Cognitive':ab,ti OR 'Mild Cognitive Impairment':ab,ti OR 'Cognitive Impairment, Mild':ab,ti OR 'Cognitive Impairments, Mild':ab,ti OR 'Impairment, Mild Cognitive':ab,ti OR 'Impairments, Mild Cognitive':ab,ti OR 'Mild Cognitive Impairments':ab,ti OR 'Cognitive Decline':ab,ti OR 'Cognitive Declines':ab,ti OR 'Decline, Cognitive':ab,ti OR 'Declines, Cognitive':ab,ti OR 'Mental Deterioration':ab,ti OR 'Deterioration, Mental':ab,ti OR 'Deteriorations, Mental':ab,ti OR 'Mental Deteriorations':ab,ti

'Exercises':ab,ti OR 'Physical Activity':ab,ti OR 'Activities, Physical':ab,ti OR 'Activity, Physical':ab,ti OR 'Physical Activities':ab,ti OR 'Exercise, Physical':ab,ti OR 'Exercises, Physical':ab,ti OR 'Physical Exercise':ab,ti OR 'Physical Exercises':ab,ti OR 'Acute Exercise':ab,ti OR 'Acute Exercises':ab,ti OR 'Isometric Exercise':ab,ti OR 'Exercise, Aerobic':ab,ti OR 'Aerobic Exercise':ab,ti OR 'Aerobic Exercises':ab,ti OR 'Exercises, Aerobic':ab,ti OR 'Exercise Training':ab,ti OR 'Exercise Trainings':ab,ti OR 'Training, Exercise':ab,ti OR 'Trainings, Exercise':ab,ti OR 'Yoga':ab,ti OR 'Tai Chi Chuan':ab,ti OR 'Running':ab,ti OR 'Jogging':ab,ti OR 'Walking':ab,ti OR 'Dancing':ab,ti OR 'Swimming':ab,ti OR 'Cycling':ab,ti OR 'Rope skipping':ab,ti

'randomized controlled trial':ab,ti OR 'randomized':ab,ti OR 'placebo':ab,ti OR 'RCT':ab,ti

**Web of science Search Strategy:**

TS=(Cognitive Dysfunction OR Cognitive Dysfunctions OR Dysfunction, Cognitive OR Dysfunctions, Cognitive OR Cognitive Impairments OR Cognitive Impairment OR Impairment, Cognitive OR Impairments, Cognitive OR Cognitive Disorder OR Cognitive Disorders OR Disorder, Cognitive OR Disorders, Cognitive OR Mild Cognitive Impairment OR Cognitive Impairment, Mild OR Cognitive Impairments, Mild OR Impairment, Mild Cognitive OR Impairments, Mild Cognitive OR Mild Cognitive Impairments OR Cognitive Decline OR Cognitive Declines OR Decline, Cognitive OR Declines, Cognitive OR Mental Deterioration OR Deterioration, Mental OR Deteriorations, Mental OR Mental Deteriorations )

TS=(Exercise OR Exercises OR Physical Activity OR Activities, Physical OR Activity, Physical OR Physical Activities OR Exercise, Physical OR Exercises, Physical OR Physical Exercise OR Physical Exercises OR Acute Exercise OR Acute Exercises OR Isometric Exercise OR Exercise, Aerobic OR Aerobic Exercise OR Aerobic Exercises OR Exercises, Aerobic OR Exercise Training OR Exercise Trainings OR Training, Exercise OR Trainings, Exercise OR Yoga OR Tai Chi Chuan OR Running OR Jogging OR Walking OR Dancing OR Swimming OR Cycling OR Rope skipping )

TS=(randomized controlled trial OR randomized OR placebo OR RCT)

**Cochrane libarary Search Strategy:**

**Participants Free terms:** (Cognitive Dysfunctions):ab,ti,kw OR (Dysfunction, Cognitive):ab,ti,kw OR (Dysfunctions, Cognitive):ab,ti,kw OR (Cognitive Impairments):ab,ti,kw OR (Cognitive Impairment):ab,ti,kw OR (Impairment, Cognitive):ab,ti,kw OR (Impairments, Cognitive):ab,ti,kw OR (Cognitive Disorder):ab,ti,kw OR (Cognitive Disorders):ab,ti,kw OR (Disorder, Cognitive):ab,ti,kw OR (Disorders, Cognitive):ab,ti,kw OR (Mild Cognitive Impairment):ab,ti,kw OR (Cognitive Impairment, Mild):ab,ti,kw OR (Cognitive Impairments, Mild):ab,ti,kw OR (Impairment, Mild Cognitive):ab,ti,kw OR (Impairments, Mild Cognitive):ab,ti,kw OR (Mild Cognitive Impairments):ab,ti,kw OR (Cognitive Decline):ab,ti,kw OR (Cognitive Declines):ab,ti,kw OR (Decline, Cognitive):ab,ti,kw OR (Declines, Cognitive):ab,ti,kw OR (Mental Deterioration):ab,ti,kw OR (Deterioration, Mental):ab,ti,kw OR (Deteriorations, Mental):ab,ti,kw OR (Mental Deteriorations):ab,ti,kw

**Intervention Free terms:** (Exercises):ab,ti,kw OR (Physical Activity):ab,ti,kw OR (Activities, Physical):ab,ti,kw OR (Activity, Physical):ab,ti,kw OR (Physical Activities):ab,ti,kw OR (Exercise, Physical):ab,ti,kw OR (Exercises, Physical):ab,ti,kw OR (Physical Exercise):ab,ti,kw OR (Physical Exercises):ab,ti,kw OR (Acute Exercise):ab,ti,kw OR (Acute Exercises):ab,ti,kw OR (Isometric Exercise):ab,ti,kw OR (Exercise, Aerobic):ab,ti,kw OR (Aerobic Exercise):ab,ti,kw OR (Aerobic Exercises):ab,ti,kw OR (Exercises, Aerobic):ab,ti,kw OR (Exercise Training):ab,ti,kw OR (Exercise Trainings):ab,ti,kw OR (Training, Exercise):ab,ti,kw OR (Trainings, Exercise):ab,ti,kw OR (Yoga):ab,ti,kw OR (Tai Chi Chuan):ab,ti,kw OR (Running):ab,ti,kw OR (Jogging):ab,ti,kw OR (Walking):ab,ti,kw OR (Dancing):ab,ti,kw OR (Swimming):ab,ti,kw OR (Cycling):ab,ti,kw OR (Rope skipping):ab,ti,kw

**Study Design Types Free terms:** randomized controlled trial,ti,kw OR (randomized):ab,ti,kw OR (placebo):ab,ti,kw OR (RCT):ab,ti,kw
