# Supplementary material for: Effects of aerobic exercise interventions on cognitive function, sleep quality, and quality of life in older adults with mild cognitive impairment: a systematic review and meta-analysis
Source: Front Neurol. 2025 Dec 17;16:1693052. doi: 10.3389/fneur.2025.1693052 (PMC12753406; doi:10.3389/fneur.2025.1693052)
Supplement: Supplementary file 3 [file Table_3.DOCX]

**Filled funnel plot with pseudo 95% confidence limits**
